# Inference of Gene Flow in the Process of Speciation: An Efficient Maximum-Likelihood Method for the Isolation-with-Initial-Migration Model

**DOI:** 10.1534/genetics.116.188060

**Published:** 2017-02-13

**Authors:** Rui J. Costa, Hilde Wilkinson-Herbots

**Affiliations:** Department of Statistical Science, University College London, WC1E 6BT, United Kingdom

**Keywords:** speciation, coalescent, maximum-likelihood, gene flow, isolation

## Abstract

The isolation-with-migration (IM) model is commonly used to make inferences about gene flow during speciation, using polymorphism data. However, it has been reported that the parameter estimates obtained by fitting the IM model are very sensitive to the model’s assumptions—including the assumption of constant gene flow until the present. This article is concerned with the isolation-with-initial-migration (IIM) model, which drops precisely this assumption. In the IIM model, one ancestral population divides into two descendant subpopulations, between which there is an initial period of gene flow and a subsequent period of isolation. We derive a very fast method of fitting an extended version of the IIM model, which also allows for asymmetric gene flow and unequal population sizes. This is a maximum-likelihood method, applicable to data on the number of segregating sites between pairs of DNA sequences from a large number of independent loci. In addition to obtaining parameter estimates, our method can also be used, by means of likelihood-ratio tests, to distinguish between alternative models representing the following divergence scenarios: (a) divergence with potentially asymmetric gene flow until the present, (b) divergence with potentially asymmetric gene flow until some point in the past and in isolation since then, and (c) divergence in complete isolation. We illustrate the procedure on pairs of *Drosophila* sequences from ∼30,000 loci. The computing time needed to fit the most complex version of the model to this data set is only a couple of minutes. The R code to fit the IIM model can be found in the supplementary files of this article.

THE two-deme, isolation-with-migration (IM) model is a population genetic model in which, at some point in the past, an ancestral population divided into two subpopulations. After the division, these subpopulations exchanged migrants at a constant rate until the present. The IM model has become one of the most popular probabilistic models in use to study genetic diversity under gene flow and population structure. Although applicable to populations within species, many researchers are using it to detect gene flow between diverging populations and to investigate the role of gene flow in the process of speciation. A meta-analysis of published research articles that used the IM model in the context of speciation can be found in Pinho and Hey (2010).

Several authors have developed computational methods to fit IM models to real DNA data. Some of the most-used programs are aimed at data sets consisting of a large number of sequences from a small number of loci. This is the case of MDIV (Nielsen and Wakeley 2001), *IM* (Hey and Nielsen 2004; Hey 2005), *IMa* (Hey and Nielsen 2007), and *IMa2* (Hey 2010), which rely on Bayesian Markov chain Monte Carlo (MCMC) methods to estimate the model parameters and are computationally very intensive.

In the past decade, the availability of large data sets spanning the entire genome has increased significantly. However, the MCMC-based implementations of the IM model referred to above are computationally expensive even for small numbers of loci, and their running times increase linearly with the number of loci (Wang and Hey 2010). Fitting an IM model also provides a rather simplified picture of the divergence process, which for some research purposes is clearly insufficient (for example, if one wishes to know whether a process of sympatric speciation has been completed, or whether gene flow occurred due to secondary contact). In addition, Becquet and Przeworski (2009) and Strasburg and Rieseberg (2010) showed that inference based on the programs *IM* and *IMa* can become unreliable if any of the assumptions made about population structure, recombination, or linkage is severely violated. For these reasons, there has been a significant increase in the demand for methods that not only scale well to genome-sized data, but are also able to estimate increasingly realistic models.

To improve efficiency and scalability, one possible strategy is to work with summary statistics rather than full data patterns. The MCMC-based program MIMAR of Becquet and Przeworski (2007, 2009) uses the four summary statistics studied by Wakeley and Hey (1997) to fit the IM model, and drops the assumption of no intralocus recombination. Gutenkunst *et al.* (2009) introduced a method based on the joint sample frequency spectrum (JSFS) that is able to fit a range of demographic models incorporating multiple populations, periods of migration and admixture, splits and joins of populations, and changes in population sizes. Based on the same type of data, the more recent implementation of Kamm *et al.* (2016) can already deal with a large number of individuals and populations, but does not yet include gene flow.

Genome-scale data sets, even when stemming from just a few individuals, tend to be more informative than data sets consisting of many individuals but covering only a relatively short genomic region. In fact, as the sample size for a single locus increases, the probability that an extra sequence adds a deep (*i.e.*, informative) branch to the coalescent tree quickly becomes negligible (see for example Hein *et al.* 2005, pp. 28–29). Data sets of a small number of individuals per locus are also more suitable for likelihood-based inference: if at each locus the observation consists only of a few sequences, the coalescent process of these sequences is relatively simple and can more easily be used to derive the likelihood for the locus concerned.

Among the methods designed for whole-genome sequence data of only a few individuals are those of Mailund *et al.* (2012), Schiffels and Durbin (2014), and Steinrücken *et al.* (2015). The fact that they are designed for full polymorphism data makes these methods computationally more expensive than JSFS-based methods. However, they rely on the coalescent with recombination modeled as a hidden Markov process, *i.e.*, they are able to capture the linkage information present in the data. Presently, complex models of demographic history can already be fitted using this approach (see, for example, Steinrücken *et al.* 2015).

Arguably the only implementations that can be considered *fast* are those based on *blockwise-likelihood* methods. These implementations are also aimed at a small number of sampled individuals, and use the information in each of a large number of relatively short and well separated loci: because recombination within loci is disregarded, it is considerably easier to derive explicitly the likelihood for each locus; and because linkage between loci is assumed to be negligible, the likelihood of a data set is just the product of the likelihoods for the individual loci.

Blockwise-likelihood methods for the standard two-deme IM model have been developed, for example, by Wilkinson-Herbots (2008) and Wang and Hey (2010), for pairs of DNA sequences at a large number of independent loci, and by Lohse *et al.* (2011) and Andersen *et al.* (2014) for larger numbers of sequences at each locus. Lohse *et al.* (2011) also developed a more general Laplace-transform method to calculate blockwise likelihoods for a range of demographic scenarios, which was further extended and efficiently automated in Lohse *et al.* (2016). Zhu and Yang (2012) developed an implementation, based on triplets of sequences, of an IM model with three species with known phylogeny and symmetric migration between two of them.

Some authors have focused on blockwise-likelihood methods for models of divergence that drop the assumption of constant gene flow until the present, and which are therefore more realistic in the context of speciation. In particular, Innan and Watanabe (2006) considered a model in which the level of gene flow between two subpopulations gradually decreases until they become completely isolated from each other. Their calculation of the likelihood of the number of nucleotide differences between pairs of sequences relies on the numerical computation of the coalescence time density at different points in time, which can be computationally expensive. IM models in which gene flow is allowed to cease at some point in the past—hereafter referred to as isolation-with-initial-migration (IIM) models—have also been considered by, for example, Teshima and Tajima (2002), Becquet and Przeworski (2009), Mailund *et al.* (2012), Wilkinson-Herbots (2012), and Lohse *et al.* (2015).

In the present article, we apply matrix eigen-decomposition techniques to expand on the work of Wilkinson-Herbots (2012) on the IIM model, who derived explicit formulas for the distribution of the coalescence time of a pair of sequences, and the distribution of the number of nucleotide differences between them. These analytic results enable a very fast computation of the likelihood under an IIM model, given a data set consisting of observations on pairs of sequences at a large number of independent loci (Lohse *et al.* 2015; Wilkinson-Herbots 2015; Janko *et al.* 2016). However, for mathematical reasons, this work adopted two biologically unrealistic assumptions which may affect the reliability of estimates: symmetric migration and equal subpopulation sizes during the migration period.

Here, we study a more general IIM model which allows for asymmetric gene flow during the migration period. It also allows for unequal subpopulation sizes during gene flow, as well as during the isolation stage. Both this model and other simpler models studied in this article assume haploid DNA sequences, which accumulate mutations according to the infinite-sites assumption (Watterson 1975). An extension to the Jukes–Cantor model of mutation is feasible but beyond the scope of this article.

We first describe an efficient method to compute the likelihood of a set of observations on the number of nucleotide differences between pairs of sequences, where each pair comes from a different locus and where we assume free recombination between loci and no recombination within loci. As our method uses an explicit expression for the likelihood, it is very fast, and efficient enough to easily deal with asymmetric bidirectional gene flow, unequal population sizes, mutation rate heterogeneity, and large numbers of mutations. Second, we illustrate how to use this method to fit the IIM model to real data. The data set of *Drosophila* sequences from Wang and Hey (2010), containing over 30,000 observations (*i.e.*, loci), is used for this purpose. Finally we demonstrate, using this data set, how different models representing different evolutionary scenarios can be compared using likelihood-ratio tests. More specifically, we compare three main scenarios: (a) divergence without gene flow; (b) divergence with potentially asymmetric gene flow until the present; and (c) divergence with potentially asymmetric gene flow until some time in the past, and in isolation since then.

## Methods

**Figure 1:**
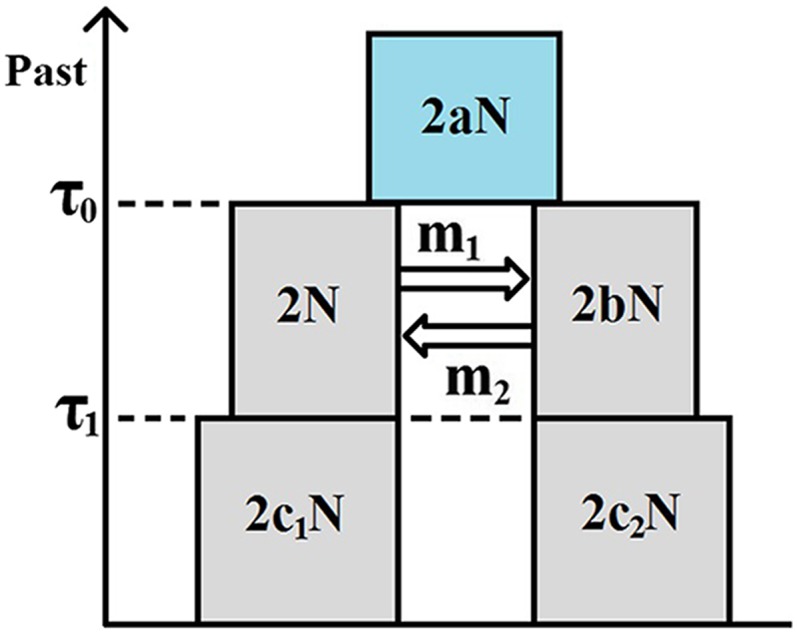
The IIM model. The left-hand-side subpopulation is subpopulation 1; the right-hand-side subpopulation is subpopulation 2.

For the purposes of the present article, and from a forward-in-time perspective, the IM model makes the following assumptions: (a) until time $\tau_{0}$ ago $\left(\tau_{0}>0\right),$ a population of DNA sequences from a single locus followed a Wright–Fisher haploid model (Fisher 1930; Wright 1931); and (b) at time $\tau_{0}$ ago, this ancestral population split into two Wright–Fisher subpopulations with constant gene flow between them. If we take an IM model and add the assumption that, at time $\tau_{1}$ ago $\left(0<\tau_{1}<\tau_{0}\right),$ gene flow ceased, we get an IIM model. Figure 1 illustrates the fullest IIM model dealt with in this article.

In the IIM model of Figure 1, the population sizes are given inside the boxes, in units of DNA sequences. All population sizes are assumed constant and strictly positive. The parameters *a*, *b*, $c_{1},$ and $c_{2}$ indicate the relative size of each population with respect to subpopulation 1 during the migration stage. For example, if $2N_{\mathrm{anc}}$ is the number of sequences in the ancestral population, then $a=2N_{\mathrm{anc}}/2N.$ Between times $\tau_{0}$ and $\tau_{1}$ ago (two time parameters in units of 2N generations), there is gene flow between the subpopulations: in each generation, a fraction $m_{i}$ of subpopulation *i* are immigrants from subpopulation *j* (i,j∈{1,2} with i≠j), *i.e.*, $m_{i}$ is the migration rate per generation from subpopulation *i* to subpopulation *j* backward in time. Within each subpopulation, reproduction follows the neutral Wright–Fisher model and, in each generation, restores the subpopulations to their original sizes, *i.e.*, reproduction undoes any decrease or increase in size caused by gene flow.

Under the IIM model, the genealogy of a sample of two DNA sequences from the present subpopulations can be described by successive Markov chains, working backward in time. We will define these in the simplest possible way, using the smallest state space necessary for the derivation of the coalescence time distribution. Hence, during the isolation stage (until time $\tau_{1}$ into the past) and the migration stage (between $\tau_{1}$ and $\tau_{0}),$ the process can only be in state 1—both lineages in subpopulation 1, state 2—both lineages in subpopulation 2, state 3—one lineage in each subpopulation, or state 4—in which lineages have coalesced. After $\tau_{0},$ the lineages have either coalesced already—state 4, or have not—state 0. Only states 1, 2, and 3 can be initial states, according to whether we sample two sequences from subpopulation 1, two sequences from subpopulation 2, or one sequence from each subpopulation. When the genealogical process starts in state *i* (with i∈{1,2,3}), the time until the most recent common ancestor of the two sampled sequences is denoted $T^{\left(i\right)},$ whereas $S^{\left(i\right)}$ denotes the number of nucleotide differences between them.

If time is measured in units of 2N generations and *N* is large, the genealogical process is well approximated by a succession of three continuous-time Markov chains; one for each stage of the IIM model (Kingman 1982a,b; Notohara 1990). We refer to this stochastic process in continuous time as the *coalescent* under the IIM model. During the isolation stage, the approximation is by a Markov chain defined by the generator matrix(1)
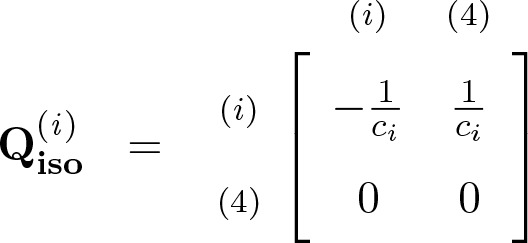
with i∈{1,2} being the initial state (Kingman 1982a,b). If 3 is the initial state, the lineages cannot coalesce before $\tau_{1}.$ During the ancestral stage, the genealogical process is approximated by a Markov chain with generator matrix(2)
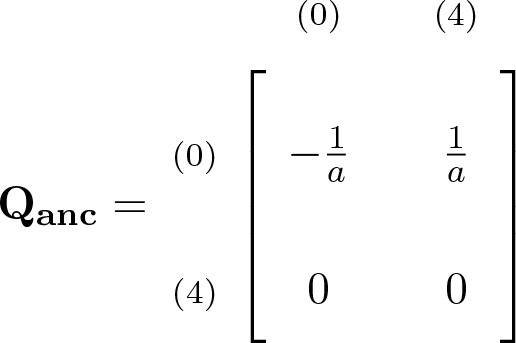
(Kingman 1982a,b). In between, during the migration stage, the approximation is by a Markov chain with generator matrix(3)
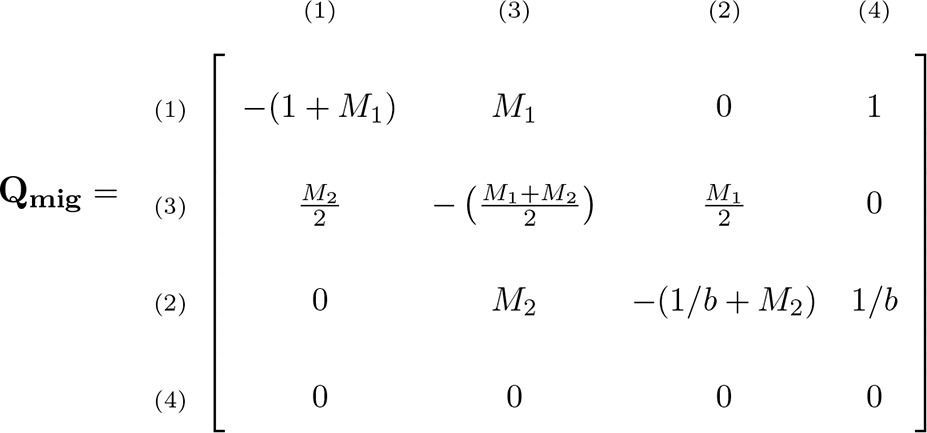
(Notohara 1990). In this matrix, $M_{i}/2=2Nm_{i}$ represents the rate of migration (in continuous time) of a single sequence when in subpopulation *i*. The rates of coalescence for two lineages in subpopulation 1 or 2 are 1 and 1/b, respectively. Note that state 3 corresponds to the second row and column, and state 2 to the third row and column. This swap was dictated by mathematical convenience: the matrix $Q_{\mathrm{mig}}$ should be as symmetric as possible because this facilitates a proof in the next section.

### Distribution of the time until coalescence under bidirectional gene flow (*M*_1_ > 0, *M*_2_ > 0)

To find $f_{T}^{\left(i\right)},$ the density of the coalescence time $T^{\left(i\right)}$ of two lineages under the IIM model, given that the process starts in state *i* and there is gene flow in both directions, we consider separately the three Markov chains mentioned above. We let $T_{\mathrm{iso}}^{\left(i\right)}$ (i∈{1,2}), $T_{\mathrm{mig}}^{\left(i\right)}$ (i∈{1,2,3}), and $T_{\mathrm{anc}}^{\left(0\right)}$ denote the times until absorption of the time-homogeneous Markov chains defined by the generator matrices $Q_{iso}^{\left(i\right)},$ $Q_{mig},$ and $Q_{anc},$ respectively. Furthermore, we let the corresponding probability density functions (PDFs) [or cumulative distribution functions (CDFs)] be denoted by $f_{\mathrm{iso}}^{\left(i\right)},$ $f_{\mathrm{mig}}^{\left(i\right)},$ and $f_{\mathrm{anc}}^{\left(0\right)}$ (or $F_{\mathrm{iso}}^{\left(i\right)},$ $F_{\mathrm{mig}}^{\left(i\right)},$ and $F_{\mathrm{anc}}^{\left(0\right)}).$ Then, $f_{T}^{\left(i\right)}$ can be expressed in terms of the distribution functions just mentioned:

$$f_{T}^{\left(i\right)}\left(t\right)=\{\begin{array}{ll} f_{\mathrm{iso}}^{\left(i\right)}\left(t\right) & \text{for }0\leq t\leq \tau_{1},\\ \left[1-F_{\mathrm{iso}}^{\left(i\right)}\left(\tau_{1}\right)\right]f_{\mathrm{mig}}^{\left(i\right)}\left(t-\tau_{1}\right) & \text{for }\tau_{1}<t\leq \tau_{0},\\ \left[1-F_{\mathrm{iso}}^{\left(i\right)}\left(\tau_{1}\right)\right]\left[1-F_{\mathrm{mig}}^{\left(i\right)}\left(\tau_{0}-\tau_{1}\right)\right]f_{\mathrm{anc}}^{\left(0\right)}\left(t-\tau_{0}\right) & \text{for }\tau_{0}<t<\infty ,\\ 0 & \text{otherwise,} \end{array}$$ (4)

for i∈{1,2}. If 3 is the initial state,

$$f_{T}^{\left(3\right)}\left(t\right)=\{\begin{array}{ll} f_{\mathrm{mig}}^{\left(3\right)}\left(t-\tau_{1}\right) & \text{for }\tau_{1}<t\leq \tau_{0},\\ \left[1-F_{\mathrm{mig}}^{\left(3\right)}\left(\tau_{0}-\tau_{1}\right)\right]f_{\mathrm{anc}}^{\left(0\right)}\left(t-\tau_{0}\right) & \text{for }\tau_{0}<t<\infty ,\\ 0 & \text{otherwise}. \end{array}$$ (5)

The important conclusion to draw from these considerations is that to find the distribution of the coalescence time under the IIM model, we only need to find the distributions of the absorption times under the simpler processes just defined.

A Markov process defined by the matrix $Q_{anc},$ and starting in state 0, is simply Kingman’s coalescent (Kingman 1982a,b). For such a process, the distribution of the coalescence time is exponential, with rate equal to the inverse of the relative population size:

$$f_{\mathrm{anc}}^{\left(0\right)}\left(t\right)=\frac{1}{a}e^{-\left(1/a\right)t},\;\;0\leq t<\infty .$$ (6)

A Markov process defined by $Q_{iso}^{\left(i\right)},$ i∈{1,2}, is again Kingman’s coalescent, so

$$f_{\mathrm{iso}}^{\left(i\right)}\left(t\right)=\frac{1}{c_{i}}e^{-\left(1/c_{i}\right)t},\;\;0\leq t<\infty .$$ (7)

Finally, with respect to the “structured” coalescent process defined by the matrix $Q_{mig},$ we prove in Appendix A that, for i∈{1,2,3},

$$f_{\mathrm{mig}}^{\left(i\right)}\left(t\right)=-\sum_{j=1}^{3}V_{ij}^{-1}V_{j4}\lambda_{j}e^{-\lambda_{j}t},$$ (8)

where $V_{ij}$ is the (i,j) entry of the (nonsingular) matrix V, whose rows are the left eigenvectors of $Q_{mig}.$ The (i,j) entry of the matrix $V^{-1}$ is denoted by $V_{ij}^{-1}.$ The $\lambda_{j}$ (j∈{1,2,3}) are the absolute values of those eigenvalues of $Q_{mig}$ which are strictly negative (the remaining one is zero). Since the $\lambda_{j}$ are real and strictly positive, the density function of $T_{\mathrm{mig}}^{\left(i\right)}$ is a linear combination of exponential densities.

Substituting the PDFs from Equations 6, 7, and 8 into the Equations 4 and 5, and denoting by A the three-by-three matrix with entries $A_{ij}=-V_{ij}^{-1}V_{j4},$ we obtain

$$f_{T}^{\left(i\right)}\left(t\right)=\{\begin{array}{ll} \frac{1}{c_{i}}e^{-\frac{1}{c_{i}}t} & \text{for }0\leq t\leq \tau_{1},\\ e^{-\frac{1}{c_{i}}\tau_{1}}\sum_{j=1}^{3}A_{ij}\lambda_{j}e^{-\lambda_{j}\left(t-\tau_{1}\right)} & \text{for }\tau_{1}<t\leq \tau_{0},\\ e^{-\frac{1}{c_{i}}\tau_{1}}\sum_{j=1}^{3}A_{ij}e^{-\lambda_{j}\left(\tau_{0}-\tau_{1}\right)}\frac{1}{a}e^{-\frac{1}{a}\left(t-\tau_{0}\right)} & \text{for }\tau_{0}<t<\infty ,\\ 0 & \text{otherwise}, \end{array}$$ (9)

for i∈{1,2}, and

$$f_{T}^{\left(3\right)}\left(t\right)=\{\begin{array}{ll} \sum_{j=1}^{3}A_{3j}\lambda_{j}e^{-\lambda_{j}\left(t-\tau_{1}\right)} & \text{for }\tau_{1}<t\leq \tau_{0},\\ \sum_{j=1}^{3}A_{3j}e^{-\lambda_{j}\left(\tau_{0}-\tau_{1}\right)}\frac{1}{a}e^{-\frac{1}{a}\left(t-\tau_{0}\right)} & \text{for }\tau_{0}<t<\infty ,\\ 0 & \text{otherwise}. \end{array}$$ (10)

If $M_{1}=M_{2}$ and b=1 (*i.e.*, in the case of symmetric gene flow and equal subpopulation sizes during the gene flow period), results 9 and 10 above simplify to the corresponding results in Wilkinson-Herbots (2012)—in this case, the coefficient $A_{i3}$ in the linear combination is zero for i∈{1,2,3}.

### Distribution of the time until coalescence under unidirectional gene flow, and in the absence of gene flow

If either $M_{1}$ or $M_{2}$ is equal to zero, or if both are equal to zero, the above derivation of $f_{\mathrm{mig}}^{\left(i\right)}$ is no longer applicable, as the similarity transformation in *Part (ii)* of the proof (Appendix A) is no longer defined (see the denominators in some entries of the matrix **D**). In this section, we derive $f_{\mathrm{mig}}^{\left(i\right)},$ the density of the absorption time of the Markov chain defined by the matrix $Q_{mig}$ given in Equation 3, starting from state i, when one or both the migration rates are zero. Again, this is all we need to fill in Equations 4 and 5 and obtain the distribution of the coalescence time of a pair of DNA sequences under the IIM model. Having gene flow in just one direction considerably simplifies the coalescent. For this reason, we resort to moment-generating functions (MGFs), instead of eigen-decomposition, and derive fully explicit PDFs.

Let $T_{\mathrm{mig}}^{\left(i\right)}$ again be defined as the absorption time of the Markov chain generated by $Q_{mig},$ now with $M_{1}=0$ and $M_{2}>0,$ given that the initial state is i∈{1,2,3}. We condition on the state of the coalescent after the first transition to obtain the following system of equations for the MGF of $T_{\mathrm{mig}}^{\left(i\right)},$ where *s* denotes a dummy variable:

$$\begin{array}{l} \text{E}\left\{\exp\left[-sT_{\mathrm{mig}}^{\left(1\right)}\right]\right\}=\left(\frac{1}{1+s}\right)\text{E}\left\{\exp\left[-sT_{\mathrm{mig}}^{\left(2\right)}\right]\right\}=\left(\frac{M_{2}}{1/b+M_{2}+s}\right)\text{E}\left\{\exp\left[-sT_{\mathrm{mig}}^{\left(3\right)}\right]\right\}+\left(\frac{1/b}{1/b+M_{2}+s}\right)\\ \text{E}\left\{\exp\left[-sT_{\mathrm{mig}}^{\left(3\right)}\right]\right\}=\left(\frac{M_{2}}{M_{2}+2s}\right)\text{E}\left\{\exp\left[-sT_{\mathrm{mig}}^{\left(1\right)}\right]\right\} \end{array}$$

(see also more general equations in Wilkinson-Herbots 1998 and Lohse *et al.* 2011). Solving this system of equations and applying a partial fraction decomposition (analogous to the work done in Griffiths 1981 and Nath and Griffiths 1993, for the case of symmetric migration and equal population sizes), the distributions of $T_{\mathrm{mig}}^{\left(1\right)},$ $T_{\mathrm{mig}}^{\left(2\right)},$ and $T_{\mathrm{mig}}^{\left(3\right)}$ can be expressed as linear combinations of exponential distributions:

$$\begin{array}{c} \text{E}\left\{\exp\left[-sT_{\mathrm{mig}}^{\left(1\right)}\right]\right\}=\left(\frac{1}{1+s}\right)\\ \text{E}\left\{\exp\left[-sT_{\mathrm{mig}}^{\left(2\right)}\right]\right\}=\left(\frac{M_{2}}{1/b+M_{2}+s}\right)\left(\frac{M_{2}}{M_{2}+2s}\right)\left(\frac{1}{1+s}\right)+\left(\frac{1/b}{1/b+M_{2}+s}\right)\\ =\left[\frac{bM_{2}^{2}}{\left(M_{2}-2\right)\left(1-b+bM_{2}\right)}\right]\left(\frac{1}{1+s}\right)+\left[\frac{4bM_{2}}{\left(2-M_{2}\right)\left(2+bM_{2}\right)}\right]\left(\frac{M_{2}}{M_{2}+2s}\right)\\ +\left[\frac{1/b}{1/b+M_{2}}+\frac{b^{2}M_{2}^{2}}{\left(2+bM_{2}\right)\left(1-b+bM_{2}\right)\left(1/b+M_{2}\right)}\right]\left(\frac{1/b+M_{2}}{1/b+M_{2}+s}\right)\\ \text{E}\left\{\exp\left[-sT_{\mathrm{mig}}^{\left(3\right)}\right]\right\}=\left(\frac{M_{2}}{M_{2}+2s}\right)\left(\frac{1}{1+s}\right)\\ =\left(\frac{M_{2}}{M_{2}-2}\right)\left(\frac{1}{1+s}\right)+\left(\frac{2}{2-M_{2}}\right)\left(\frac{M_{2}}{M_{2}+2s}\right). \end{array}$$

Thus we obtain the following PDFs:

$$\begin{array}{c} f_{\mathrm{mig}}^{\left(1\right)}\left(t\right)=e^{-t}\\ f_{\mathrm{mig}}^{\left(2\right)}\left(t\right)=\left[\frac{bM_{2}^{2}}{\left(M_{2}-2\right)\left(1-b+bM_{2}\right)}\right]e^{-t}+\left[\frac{4bM_{2}}{\left(2-M_{2}\right)\left(2+bM_{2}\right)}\right]\frac{M_{2}}{2}e^{-\frac{M_{2}}{2}t}\\ +\left[\frac{1}{1+bM_{2}}+\frac{b^{2}M_{2}^{2}}{\left(2+bM_{2}\right)\left(1-b+bM_{2}\right)\left(1/b+M_{2}\right)}\right]\left(\frac{1}{b}+M_{2}\right)e^{-\left(1/b+M_{2}\right)t}\\ f_{\mathrm{mig}}^{\left(3\right)}\left(t\right)=\left(\frac{M_{2}}{M_{2}-2}\right)e^{-t}+\left(\frac{2}{2-M_{2}}\right)\frac{M_{2}}{2}e^{-\frac{M_{2}}{2}t} \end{array}$$

for t>0.

The PDF of the coalescence time of a pair of DNA sequences under an IIM model with $M_{1}=0$ and $M_{2}>0$ can now be easily derived by comparing the above expressions with Equation 8: $f_{T}^{\left(i\right)}\left(t\right)$ is given by Equations 9 and 10 above, but now with

$$\lambda =\left[\begin{array}{ccc} 1 & \frac{M_{2}}{2} & \frac{1}{b}+M_{2} \end{array}\right],$$

and

$$A=\left[\begin{array}{ccc} 1 & 0 & 0\\ \frac{bM_{2}^{2}}{\left(M_{2}-2\right)\left(1-b+bM_{2}\right)} & \frac{4bM_{2}}{\left(2-M_{2}\right)\left(2+bM_{2}\right)} & \frac{1}{1+bM_{2}}+\frac{b^{2}M_{2}^{2}}{\left(2+bM_{2}\right)\left(1-b+bM_{2}\right)\left(1/b+M_{2}\right)}\\ \frac{M_{2}}{M_{2}-2} & \frac{2}{2-M_{2}} & 0 \end{array}\right].$$

In the opposite case of unidirectional migration $\left(M_{1}>0,M_{2}=0\right),$ we obtained the distribution of the time until coalescence using essentially the same procedure as described above. In addition, for $M_{1}=M_{2}=0,$ the derivation is trivial. The results for these two cases can be found in Appendix B.

### The distribution of the number *S* of segregating sites

Let $S^{\left(i\right)}$ denote the number of segregating sites in a random sample of two sequences from a given locus, when the ancestral process of these sequences follows the coalescent under the IIM model and the initial state is state *i* (i∈{1,2,3}). Recall the infinite-sites assumption and assume that the distribution of the number of mutations hitting one sequence in a single generation is Poisson with mean *μ*. As before, time is measured in units of 2N generations and we use the coalescent approximation. Given the coalescence time $T^{\left(i\right)}$ of two sequences, $S^{\left(i\right)}$ is Poisson distributed with mean $\theta T^{\left(i\right)},$ where θ=4Nμ denotes the scaled mutation rate. Since the PDF of $T^{\left(i\right)},$ $f_{T}^{\left(i\right)},$ is known, the likelihood $L^{\left(i\right)}$ of an observation from a single locus corresponding to the initial state *i* can be derived by integrating out $T^{\left(i\right)}$:

$$L^{\left(i\right)}\left(\gamma ,\theta ;s\right)=P\left[S^{\left(i\right)}=s;\gamma ,\theta \right]=\int_{0}^{\infty }P\left[S^{\left(i\right)}=s|T^{\left(i\right)}=t\right]f_{T}^{\left(i\right)}\left(t\right)\text{d}t,$$

where γ is the vector of parameters of the coalescent under the IIM model, that is, $\gamma =\left(a,b,c_{1},c_{2},\tau_{1},\tau_{0},M_{1},M_{2}\right).$ There is no need to compute this integral numerically: because $f_{T}^{\left(i\right)}$ has been expressed in terms of a piecewise linear combination of exponential or shifted exponential densities, we can use standard results for a Poisson process superimposed onto an exponential or shifted exponential distribution.

The equations 18 and 29 of Wilkinson-Herbots (2012) use this superimposition of processes to derive the distribution of *S* under a mathematically much simpler IIM model with symmetric migration and equal subpopulation sizes during the period of migration. These equations can now be adapted to obtain the probability mass function (PMF) of *S* under each of the migration scenarios dealt with in this article. The changes accommodate the fact that the density of the coalescence time during the migration stage of the model is now given by a different linear combination of exponential densities, where the coefficients in the linear combination, as well as the parameters of the exponential densities, are no longer the same. The PMF of *S* has the following general form:

$$\begin{array}{c} P\left[S^{\left(i\right)}=s\right]=\frac{{\left(c_{i}\theta \right)}^{s}}{{\left(1+c_{i}\theta \right)}^{s+1}}\left[1-e^{-\tau_{1}\left(\frac{1}{c_{i}}+\theta \right)}\sum_{l=0}^{s}\frac{{\left(\frac{1}{c_{i}}+\theta \right)}^{l}\tau_{1}^{l}}{l!}\right]\\ +e^{-\frac{1}{c_{i}}\tau_{1}}\sum_{j=1}^{3}A_{ij}\frac{\lambda_{j}\theta^{s}}{{\left(\lambda_{j}+\theta \right)}^{s+1}}[e^{-\theta \tau_{1}}\sum_{l=0}^{s}\frac{{\left(\lambda_{j}+\theta \right)}^{l}\tau_{1}^{l}}{l!}\\ -e^{-\lambda_{j}\left(\tau_{0}-\tau_{1}\right)-\theta \tau_{0}}\sum_{l=0}^{s}\frac{{\left(\lambda_{j}+\theta \right)}^{l}\tau_{0}^{l}}{l!}]\\ +\frac{e^{-\frac{1}{c_{i}}\tau_{1}-\theta \tau_{0}}{\left(a\theta \right)}^{s}}{{\left(1+a\theta \right)}^{s+1}}\left[\sum_{l=0}^{s}\frac{{\left(\frac{1}{a}+\theta \right)}^{l}\tau_{0}^{l}}{l!}\right]\sum_{j=1}^{3}A_{ij}e^{-\lambda_{j}\left(\tau_{0}-\tau_{1}\right)} \end{array}$$ (11)

for i∈{1,2}, and

$$\begin{array}{c} P\left[S^{\left(3\right)}=s\right]=\sum_{j=1}^{3}A_{3j}\frac{\lambda_{j}\theta^{s}}{{\left(\lambda_{j}+\theta \right)}^{s+1}}[e^{-\theta \tau_{1}}\sum_{l=0}^{s}\frac{{\left(\lambda_{j}+\theta \right)}^{l}\tau_{1}^{l}}{l!}\\ -e^{-\lambda_{j}\left(\tau_{0}-\tau_{1}\right)-\theta \tau_{0}}\sum_{l=0}^{s}\frac{{\left(\lambda_{j}+\theta \right)}^{l}\tau_{0}^{l}}{l!}]\\ +\frac{e^{-\theta \tau_{0}}{\left(a\theta \right)}^{s}}{{\left(1+a\theta \right)}^{s+1}}\left[\sum_{l=0}^{s}\frac{{\left(\frac{1}{a}+\theta \right)}^{l}\tau_{0}^{l}}{l!}\right]\sum_{j=1}^{3}A_{3j}e^{-\lambda_{j}\left(\tau_{0}-\tau_{1}\right)} \end{array}$$ (12)

for s∈{0,1,2,3,…}.. As defined in the *Distribution of the time until coalescence under bidirectional gene flow (M_1_ > 0*, *M_2_ > 0)* section, under bidirectional migration $\lambda =\left(\lambda_{1},\lambda_{2},\lambda_{3}\right)$ is the vector of the absolute values of the strictly negative eigenvalues of $Q_{mig}$ and $A_{ij}=-V_{ij}^{-1}V_{j4}.$ If migration occurs in one direction only, with $M_{1}=0$ and $M_{2}>0,$ the matrix A and the vector λ are those given in the *Distribution of the time until coalescence under unidirectional gene flow*, *and in the absence of gene flow* section. In the remaining cases, when $M_{1}>0$ and $M_{2}=0$ or when there is no gene flow, A and λ are given in Appendix B. In the special case of $M_{1}=M_{2}$ and b=1, Equations 11 and 12 reduce to the results of Wilkinson-Herbots (2012).

### The likelihood of a multilocus data set

Recall that, for our purposes, an observation consists of the number of nucleotide differences between a pair of DNA sequences from the same locus. To jointly estimate all the parameters of the IIM model, our method requires a large set of observations on each of the three initial states (*i.e.*, on pairs of sequences from subpopulation 1, from subpopulation 2, and from both subpopulations). To compute the likelihood of such a data set, we use the assumption that observations are independent, so we should have no more than one observation or pair of sequences per locus and there should be free recombination between loci, *i.e.*, loci should be sufficiently far apart.

Let each locus for the initial state *i* be assigned a label $j_{i}\in \left\{1_{i},2_{i},3_{i},\ldots ,J_{i}\right\},$ where $J_{i}$ is the total number of loci associated with initial state *i*. Denote by $\theta_{j_{i}}=4N\mu_{j_{i}}$ the scaled mutation rate at locus $j_{i},$ where $\mu_{j_{i}}$ is the mutation rate per sequence per generation at that locus. Let *θ* denote the average scaled mutation rate over all loci and denote by $r_{j_{i}}=\theta_{j_{i}}/\theta$ the relative mutation rate of locus $j_{i}.$ Then, $\theta_{j_{i}}=r_{j_{i}}\theta .$ If the relative mutation rates are known, we can represent the likelihood of the observation at locus $j_{i}$ simply by $L\left(\gamma ,\theta ;s_{j_{i}}\right).$ By independence, the likelihood of the data set is then given by

$$L\left(\gamma ,\theta ;s\right)=\prod_{i=1}^{3}\prod_{j_{i}=1}^{J_{i}}L\left(\gamma ,\theta ;s_{j_{i}}\right).$$ (13)

In our likelihood method, the $r_{j_{i}}$ are treated as known constants. In practice, however, the relative mutation rates at the different loci are usually estimated using outgroup sequences (Yang 2002; Wang and Hey 2010).

### Data availability

In the Supplemental Material, File S1 contains the R code to fit the IIM model (and other simpler models) to data sets consisting of observations on the number of segregating sites between pairs of DNA sequences from a large number of independent loci. File S2 contains the R code we used to simulate observations from the IIM model. File S3 contains R functions that are required by File S1 and File S2. The raw *Drosophila* sequence data used in this article were published by Wang and Hey (2010); the processed *Drosophila* data to which the models of Figure 7 were fitted are given in File S4.

## Results

### Simulated data

We generated three batches of data sets by simulation, each batch having 100 data sets. Each data set consists of thousands of independent observations, where each observation represents the number of nucleotide differences between two DNA sequences belonging to the same locus, when the genealogy of these sequences follows an IIM model. Each data set of batches 1, 2, and 3 contains 8000, 40,000, and 800,000 observations, respectively. In each data set, half of the observations correspond to initial state 3, 1/4 to initial state 1, and 1/4 to initial state 2.

The data sets shown in this section were generated using the following parameter values: a=0.75, θ=2, b=1.25, $c_{1}=1.5,$ $c_{2}=2,$ $\tau_{0}=2,$ $\tau_{1}=1,$ $M_{1}=0.5,$ and $M_{2}=0.75.$ Each observation in a data set refers to a different genetic locus *j*, and hence was generated using a different scaled mutation rate $\theta_{j}$ for that locus. For batch 1, we first fixed the average mutation rate over all sites to be θ=2. Then, a vector of 8000 relative-size scalars $r_{j}$ was randomly generated using a Gamma (15, 15) distribution. The scaled mutation rate at locus *j* was then defined to be $\theta_{j}=r_{j}\theta ,$ where $r_{j}$ denotes the relative mutation rate at locus *j*, that is, the relative size of $\theta_{j}$ with respect to the average mutation rate *θ*. All data sets in batch 1 were generated using the same vector of relative mutation rates. The generation of the mutation rates $\theta_{j}$ used in batches 2 and 3 was carried out following the same procedure.

When fitting the IIM model to data sets generated in this manner, the relative mutation rates $r_{j}$ are included as known constants in the log-likelihood function to be maximized. So, as far as mutation rates are concerned, only the average over all loci is estimated (*i.e.*, the parameter *θ*). To increase the robustness and performance of the fitting procedure (see also Wilkinson-Herbots 2015, and the references therein), we found the maximum-likelihood estimates for a reparameterized model with parameters *θ*, $\theta_{a}=\theta a,$ $\theta_{b}=\theta b,$ $\theta_{c_{1}}=\theta c_{1},$ $\theta_{c_{2}}=\theta c_{2},$ $V=\theta \left(\tau_{0}-\tau_{1}\right),$ $T_{1}=\theta \tau_{1},$ $M_{1},$ and $M_{2}.$

**Figure 2:**
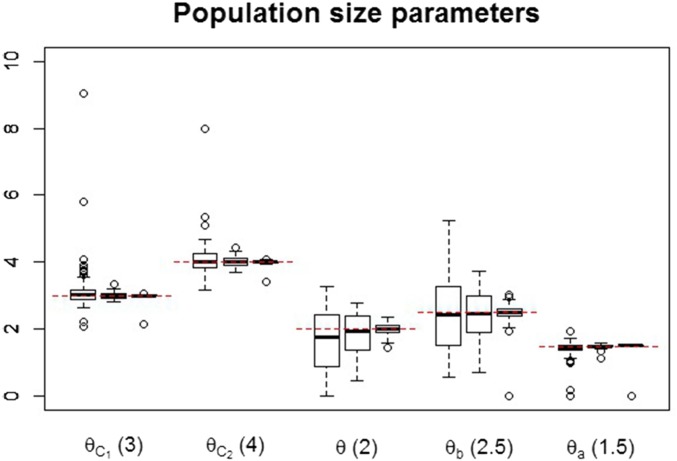
Estimates of population size parameters for simulated data. For each parameter, the estimates shown on the left, center, and right-hand-side boxplots are based on sample sizes of 8000, 40,000, and 800,000 loci, respectively. The values stated in parentheses are the true parameter values used to generate the data. Horizontal dashed lines indicate the true parameter values for each group of boxplots.

**Figure 3:**
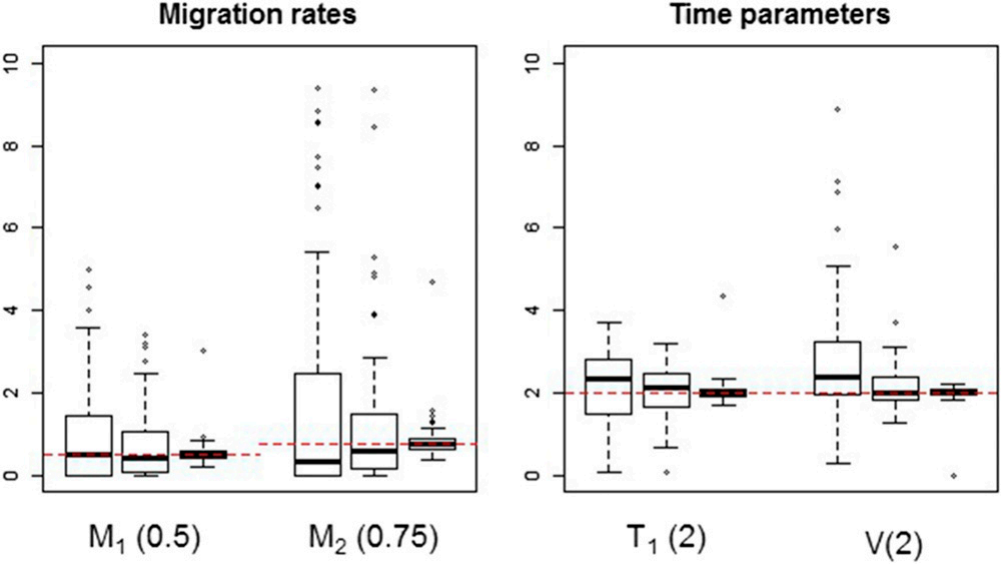
Estimates of migration rates and time parameters for simulated data. For each parameter, the estimates shown on the left, center, and right-hand-side boxplots are based on sample sizes of 8000, 40,000, and 800,000 loci, respectively. The values stated in parentheses are the true parameter values used to generate the data. Horizontal dashed lines indicate the true parameter values for each group of boxplots.

The boxplots of the maximum-likelihood estimates obtained for the three batches of simulated data are shown in Figure 2 and Figure 3. For each parameter, the boxplots on the left, center, and right-hand side refer to batches 1, 2, and 3, respectively. From the boxplots of time and population size parameters, it is seen that the estimates are centered around the true parameter values. Estimates for the migration rates are skewed to the right for batches 1 and 2, possibly because the true parameter values for these rates are closer to the boundary (zero) than the ones for population sizes and splitting times. For all types of parameters, increasing the sample size will decrease the variance of the maximum-likelihood estimator, as would be expected from using the correct expressions for the likelihood. In the case of the migration rate parameters, increasing the sample size eliminates most of the skewness.

**Figure 4:**
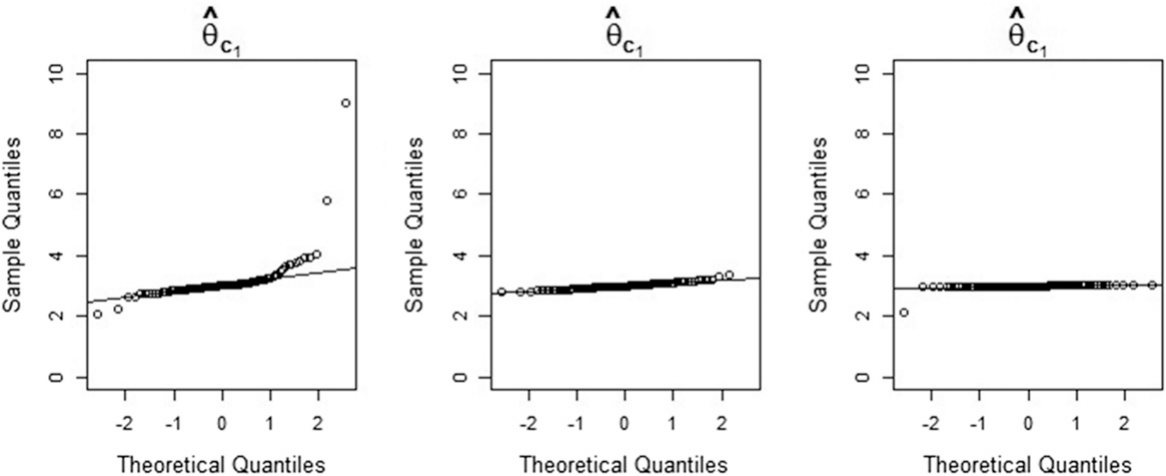
Q-Q plots of maximum-likelihood estimates of the parameter $\theta_{c_{1}}$ obtained from simulated data, against the theoretical quantiles of the standard normal distribution. The estimates shown in the left-hand-side, center, and right-hand-side Q-Q plots are based on sample sizes of 8000, 40,000, and 800,000 loci, respectively. In the central Q-Q plot, one outlier with a value above 10 is not shown.

**Figure 5:**
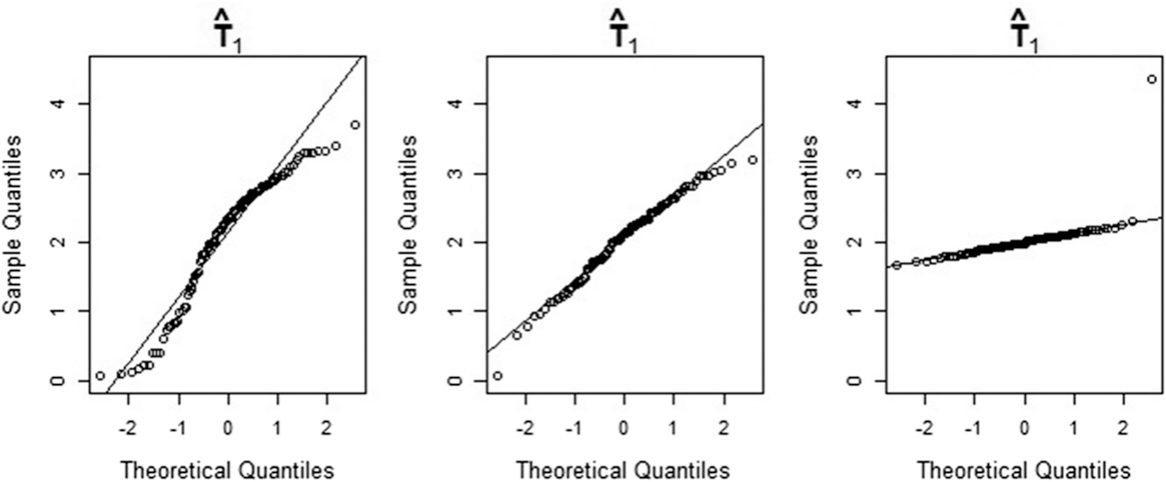
Q-Q plots of maximum-likelihood estimates of the parameter $T_{1}$ obtained from simulated data, against the theoretical quantiles of the standard normal distribution. The estimates shown in the left-hand-side, center, and right-hand-side Q-Q plots are based on sample sizes of 8000, 40,000, and 800,000 loci, respectively.

**Figure 6:**
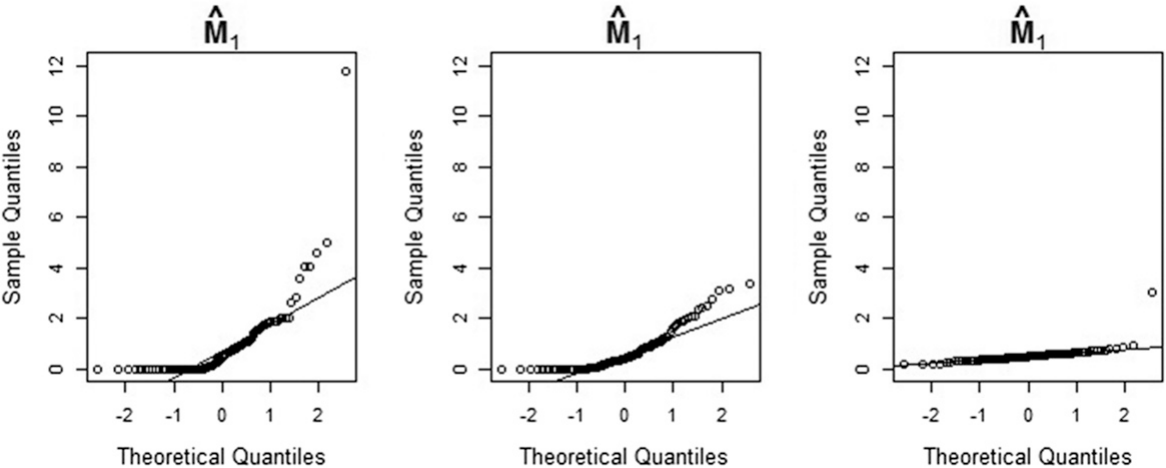
Q-Q plots of maximum-likelihood estimates of the parameter $M_{1}$ obtained from simulated data, against the theoretical quantiles of the standard normal distribution. The estimates shown in the left-hand-side, center, and right-hand-side Q-Q plots are based on sample sizes of 8000, 40,000, and 800,000 loci, respectively.

The three quantile-quantile (Q-Q) plots in Figure 4 show the sample quantiles of the maximum-likelihood estimates of $\theta_{c_{1}}$ (a size parameter) obtained from simulated data, plotted against the theoretical quantiles of the standard normal distribution. Figure 5 and Figure 6 show the corresponding plots for $T_{1}$ (a time parameter) and $M_{1}$ (a migration parameter). In each figure, the left-hand side, center, and right-hand-side Q-Q plots are based on simulation batches 1, 2, and 3, respectively. It is clear from Figure 4, Figure 5, and Figure 6 that the distributions of the maximum-likelihood estimates of $\theta_{c_{1}},$ $T_{1},$ and $M_{1}$ become increasingly Gaussian as we increase the number of observations. This is also true for the estimates of the remaining parameters (results not shown). We note also that the distributions of the time and population size estimates already have a reasonably Gaussian shape for a sample size of 8000 loci. Again, this is true for the estimates of the remaining time and size parameters as well. The lack of approximate normality of the migration rate estimates for smaller sample sizes suggests care should be taken when making inferences about these parameters—see *Notes on our method and results*.

### Drosophila DNA sequence data

#### Maximum-likelihood estimation:

To illustrate our method, we apply it to a real, multilocus data set from two closely related species of *Drosophila*: *Drosophila simulans* and *D. melanogaster*. The DNA sequence data of Wang and Hey (2010) consist of two subsets: a large subset, which we will call the “Wang subset,” containing 30,247 blocks of intergenic sequence; and a smaller subset, which we will refer to as the “Hutter subset,” consisting of 378 blocks of intergenic sequence. Loci in the Wang subset were sampled by Wang and Hey (2010) from a genome alignment of four inbred lines, two from *D. simulans*, and one from each of *D. melanogaster* and *D. yakuba*. To take into account the assumption of no recombination within loci and free recombination between loci, and based on the findings of Hey and Nielsen (2004) regarding the density of apparent recombination events in *Drosophila*, Wang and Hey (2010) chose a locus length of ∼500 bp and a space of at least 2000 bp between loci. To build the Hutter subset, they drew 378 pairs of *D. melanogaster* sequences from the data set of Hutter *et al.* (2007), which consists of 378 blocks of sequence sampled from 24 inbred lines of *D. melanogaster*, with an average locus length of 536 bp and an average distance of ∼52 kb between consecutive loci. They then joined each of these sequence pairs with their respective *D. yakuba* orthologs from the *simulans-melanogaster-yakuba* genome alignment. Our models are fitted to the *D. melanogaster* and *D. simulans* sequences from both subsets. The *D. yakuba* sequences are only used as outgroup sequences, to estimate the relative mutation rates at the different loci and to calibrate time.

Since our method uses only one pair of sequences at each of a large number of independent loci, and requires observations for all initial states, the following procedure was adopted to select a suitable set of data. According to the genome assembly they stem from, sequences in the Wang subset were given one of three possible tags: “Dsim1,” “Dsim2,” or “Dmel.” To each of the 30,247 loci in the Wang subset, we assigned a letter: loci with positions 1, 4, 7, … in the genome alignment were assigned the letter A; loci with positions 2, 5, 8, … were assigned the letter B; and loci with positions 3, 6, 9, … were assigned the letter C. A data set was then built by selecting observations corresponding to initial states 1 and 3 from the Wang subset (we used the Dsim1-Dsim2 sequences from loci A, the Dmel-Dsim1 sequences from loci B, and the Dmel-Dsim2 sequences from loci C), while observations corresponding to initial state 2 were obtained from the Hutter subset by comparing the two *D. melanogaster* sequences available at each locus.

To estimate the relative mutation rates $r_{j_{i}},$ we use the *ad hoc* approach proposed by Yang (2002), which was also used in Wang and Hey (2010) and Lohse *et al.* (2011). Estimates are computed by means of the following method-of-moments estimator:

$${\hat{r}}_{j_{i}}=\frac{J{\bar{k}}_{j_{i}}}{\sum_{m=1}^{3}\sum_{n=1}^{J_{m}}{\bar{k}}_{n_{m}}},$$ (14)

where *J* is the total number of loci, and ${\bar{k}}_{j_{i}}$ is the average of the numbers of nucleotide differences observed in pairs of one ingroup sequence and one outgroup sequence, at locus $j_{i}.$

**Figure 7:**
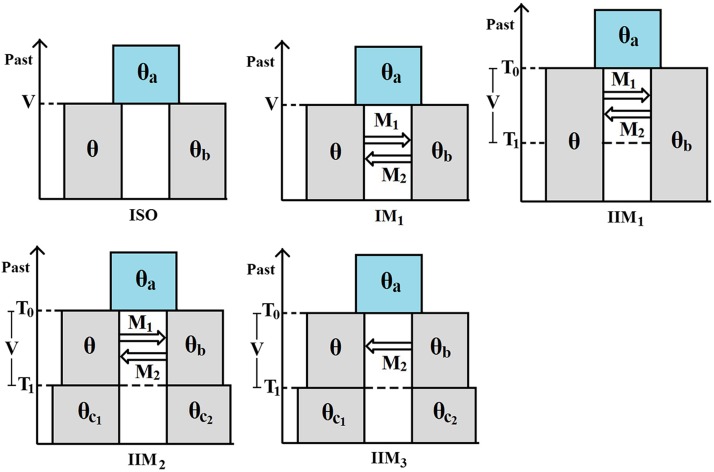
Models fitted to the data of Wang and Hey (2010): $\theta_{a}=\theta a,$ $\theta_{b}=\theta b,$ $\theta_{c_{1}}=\theta c_{1},$ $\theta_{c_{2}}=\theta c_{2},$ $V=T_{0}-T_{1}=\theta \left(\tau_{0}-\tau_{1}\right),$ and $T_{1}=\theta \tau_{1}.$

Table 1 contains the maximum-likelihood estimates for the models shown in Figure 7. Note that the parameters of time and population size have been reparameterized as in *Simulated data*, and recall that $M_{1}$ and $M_{2}$ are the scaled migration rates backward in time. In the diagrams, the left and right subpopulations represent *D. simulans* and *D. melanogaster*, respectively.

**Table 1:** Maximum-likelihood estimates and values of the maximized log-likelihood

Model	$\theta_{a}$	θ	$\theta_{b}$	$\theta_{c_{1}}$	$\theta_{c_{2}}$	$T_{1}$	V	$M_{1}$	$M_{2}$	logL(φ)
ISO	4.757	5.628	2.665	—	—	—	13.705	—	—	−90,879.14
IM_1_	3.974	5.641	2.493	—	—	—	14.965	0.000	0.053	−90,276.00
IIM_1_	3.191	5.581	2.589	—	—	6.931	9.928	0.000	0.528	−90,069.44
IIM_2_	3.273	3.357	1.929	6.623	2.647	6.930	9.778	0.000	0.223	−89,899.22
IIM_3_	3.273	3.357	1.929	6.623	2.647	6.930	9.778	—	0.223	−89,899.22

Results for the data of Wang and Hey (2010), for the models shown in Figure 7.

#### Model selection:

In this section, we use a series of likelihood-ratio tests for nested models to determine which of the models listed in Table 1 fits the data of Wang and Hey (2010) best. The use of such tests in the present situation is not entirely straightforward. We wish to apply a standard large-sample theoretical result which states that, as the number of observations increases, the distribution of the likelihood-ratio test statistic given by

D=−2log λ(s),

where

$$\lambda \left(s\right)=\frac{\underset{\varphi \in \Phi_{0}}{\sup}L\left(\varphi ;s\right)}{\underset{\varphi \in \Phi }{\sup}L\left(\varphi ;s\right)},$$ (15)

approaches a $\chi^{2}$ distribution. In Equation 15, $\Phi_{0}$ denotes the parameter space according to the null hypothesis $\left(H_{0}\right).$ This space is a proper subspace of Φ, the parameter space according to the alternative hypothesis $\left(H_{1}\right).$ The number of degrees of freedom of the limiting distribution is given by the difference between the dimensions of the two spaces. A list of sufficient regularity conditions for this result can be found, for example, in Casella and Berger (2001, p. 516). One of them is clearly not met in the present case: in the pairwise comparison of some of our models, every point of $\Phi_{0}$ is a boundary point of Φ. In other words, if $H_{0}$ is true, the vector of true parameters $\varphi *\in \Phi_{0},$ whichever it might be, is on the boundary of Φ. This irregularity is present, for example, when $M_{1}=M_{2}=0$ according to $H_{0}$ and $M_{1},$ $M_{2}\in \left[0,\infty \right)$ according to $H_{1}.$ The problem of parameters on the boundary has been the subject of articles such as Self and Liang (1987) and Kopylev and Sinha (2011). The limiting distribution of the likelihood-ratio test statistic under this irregularity has been derived in these articles, but only for very specific cases. In most of these cases, the use of the naive $\chi_{r}^{2}$ distribution, with *r* being the number of additional free parameters according to $H_{1},$ turns out to be conservative, because the correct null distribution is a mixture of $\chi_{\nu }^{2}$ distributions with ν≤r. Our analysis of the data of Wang and Hey (2010) involves two likelihood-ratio tests with parameters on the boundary (ISO *vs.* IM_1_, and IM_1_ *vs.* IIM_1_), so we need to check that the naive $\chi_{r}^{2}$ distribution is also conservative in these cases. This was verified in a short simulation study which we now describe.

**Figure 8:**
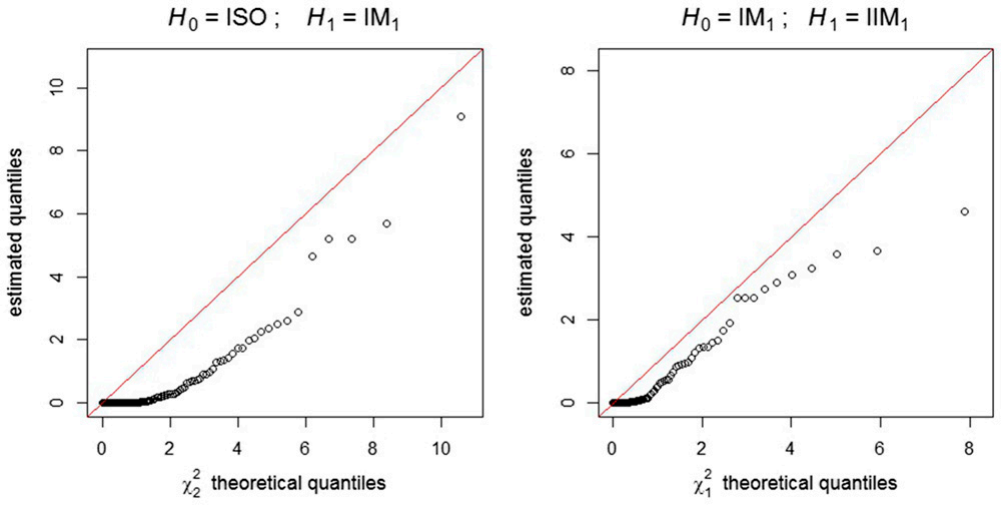
Q-Q plots of the estimated quantiles of the likelihood-ratio test statistic null distribution against the $\chi^{2}$ distribution theoretical quantiles. Left plot: $H_{0}$ = ISO model, $H_{1}$ = IM_1_ model. Right plot: $H_{0}$ = IM_1_ model, $H_{1}$ = IIM_1_ model.

We generated 100 data sets from the ISO model, each one consisting of 40,000 observations, and fitted both the ISO model $\left(H_{0}\right)$ and the IM_1_ model $\left(H_{1}\right)$ to obtain a sample of 100 realizations of the likelihood-ratio test statistic. A Q-Q plot (Figure 8, left boxplot) shows that the estimated quantiles of the null distribution are smaller than the corresponding theoretical quantiles of the $\chi^{2}$ distribution with two degrees of freedom (the difference between the dimensions of $\Phi_{0}$ and Φ in this particular case). In other words, the use of the naive $\chi^{2}$ distribution is conservative in this case. Using $\chi_{2}^{2}$ instead of the correct null distribution, at a significance level of 5%, the null hypothesis (*i.e.*, the ISO model) was falsely rejected in only 1 out of the 100 simulations performed.

A similar simulation was carried out with respect to another pair of nested models: the IM_1_ model (now as *H*_0_), in which $\tau_{1}=0,$ and the IIM_1_ model $\left(H_{1}\right),$ in which $\tau_{1}>0.$ Again, the naive $\chi^{2}$ distribution (this time with only one degree of freedom) was found to be conservative (Figure 8, right boxplot). And once more, only in 1 out of the 100 simulations performed is the null hypothesis (the IM_1_ model) falsely rejected at the 5% significance level, if $\chi_{1}^{2}$ is used instead of the correct null distribution.

To select the model that best fitted the data of Wang and Hey (2010), we performed the sequence of pairwise comparisons shown in Table 2. For any sensible significance level, this sequence of comparisons leads to the choice of IIM_2_ as the best-fitting model. In fact, assuming the naive $\chi^{2}$ as the null distribution, a significance level as low as $1.2\times 10^{-74}$ is enough to reject $H_{0}$ in each of the three tests. However, since ${\hat{M}}_{1}=0$ for this model (see Table 1), a final (backward) comparison is in order: one between IIM_2_ and IIM_3_ (which corresponds to fixing $M_{1}$ at zero in IIM_2_). The nested model in this comparison has one parameter less and, as can be seen in Table 1, has the same likelihood. So, in the end, we should prefer IIM_3_ to IIM_2_.

**Table 2:** Forward selection of the best model

$H_{0}$	$H_{1}$	−2logλ(S)	*P*-value
ISO	IM_1_	603.14	1.147E−262
IM_1_	IIM_1_	413.12	7.673E−92
IIM_1_	IIM_2_	340.44	1.187E−74

Results refer to the data of Wang and Hey (2010).

#### Confidence intervals for the selected model:

The Wald confidence intervals are straightforward to calculate whenever the vector of estimates is neither on the boundary of the model’s parameter space, nor too close to it. In that case, it is reasonable to assume that the vector of *true* parameters does not lie on the boundary either. As a consequence, the vector of maximum-likelihood estimators is consistent and its distribution will approach a multivariate Gaussian distribution as the sample size grows (see, for example, Pawitan 2001, p. 258). The confidence intervals can then be calculated using the inverted Hessian matrix.

In the case of the data of Wang and Hey (2010), the vector of estimates of the selected model (IIM_3_) is an interior point of the parameter space. Assuming that the vector of true parameters is also away from the boundary, we computed the Wald 95% confidence intervals shown in Table 3 using the inverted Hessian. In agreement with our assumption, we note that none of the confidence intervals include zero.

**Table 3:** Point estimates and confidence intervals under the model IIM_3_

Parameter	Estimate	95% confidence intervals	
		Wald	Profile likelihood
$\theta_{a}$	3.273	(3.101, 3.445)	(3.100, 3.444)
*θ*	3.357	(3.139, 3.575)	(3.097, 3.578)
$\theta_{b}$	1.929	(0.079, 3.779)	(0.672, 5.010)
$\theta_{c_{1}}$	6.623	(6.407, 6.839)	(6.415, 6.843)
$\theta_{c_{2}}$	2.647	(2.304, 2.990)	(2.331, 3.021)
$T_{1}$	6.930	(6.540, 7.320)	(6.542, 7.319)
*V*	9.778	(9.457, 10.099)	(9.456, 10.098)
$M_{2}$	0.223	(0.190, 0.256)	(0.186, 0.259)

Results refer to the data of Wang and Hey (2010).

For large sample sizes, and for true parameter values not too close to the boundary of the parameter space, the Wald intervals are both accurate and easy to compute. To check how well the Wald intervals for the IIM_3_ model fare against the more accurate (see Pawitan 2001, pp. 47–48), but also computationally more expensive, profile likelihood intervals, we included these in Table 3. The two methods yield very similar confidence intervals for all parameters except $\theta_{b}.$ The cause of this discrepancy should lie in the fact that we only had pairs of *D. melanogaster* sequences available from a few hundred loci $(\theta_{b}$ is the size of the *D. melanogaster* subpopulation during the migration stage).

#### Conversion of estimates:

The conversion of point estimates and confidence intervals to more conventional units is based on the estimates of Powell (1997) of the duration of one generation (g=0.1 years) and the speciation time between *D. yakuba* and the common ancestor of *D. simulans* and *D. melanogaster* (10 MY); see also Wang and Hey (2010) and Lohse *et al.* (2011). Using these values, we estimated *μ*, the mutation rate per locus per generation, averaged over all loci, to be $\hat{\mu }=2.31\times 10^{-7}.$

In Table 4, Table 5, and Table 6, we show the converted estimates for the best-fitting model IIM_3_. The effective population size estimates, in units of diploid individuals, are all based on estimators of the form $\hat{N}=\left(1/4\hat{\mu }\right)\times \hat{\theta }.$ For example, the estimate of the ancestral population effective size $N_{a}$ is given by $\left(1/4\hat{\mu }\right)\times {\hat{\theta }}_{a}.$ The estimates in years of the time since the onset of speciation and of the time since the end of gene flow are given by ${\hat{t}}_{0}=\left(g/2\hat{\mu }\right)\times \left({\hat{T}}_{1}+\hat{V}\right)$ and ${\hat{t}}_{1}=\left(g/2\hat{\mu }\right)\times {\hat{T}}_{1},$ respectively. With respect to gene flow, we use ${\hat{q}}_{1}=\hat{\mu }\times \left({\hat{M}}_{2}\hat{b}/\hat{\theta }\right)$ as the estimator of the *fraction* of subpopulation 1 that migrates to subpopulation 2 in each generation, forward in time; and ${\hat{s}}_{1}=\left({\hat{M}}_{2}\hat{b}/2\right)$ as the estimator of the *number* of migrant sequences from subpopulation 1 to subpopulation 2 in each generation, also forward in time.

**Table 4:** Effective population size estimates under the model IIM_3_

Population	Population size	95% confidence intervals	
		Wald	Profile likelihood
Ancestral population $\left(N_{a}\right)$	3.549	(3.362, 3.736)	(3.362, 3.735)
*D. simulans*, migration stage (*N*)	3.640	(3.404, 3.877)	(3.359, 3.880)
*D. melanogaster*, migration stage $\left(N_{b}\right)$	2.092	(0.085, 4.099)	(0.729, 5.433)
*D. simulans*, isolation stage $\left(N_{c_{1}}\right)$	7.182	(6.949, 7.415)	(6.957, 7.421)
*D. melanogaster*, isolation stage $\left(N_{c_{2}}\right)$	2.871	(2.498, 3.243)	(2.528, 3.276)

Effective population size estimates for the data of Wang and Hey (2010). Values are in millions of diploid individuals.

**Table 5:** Divergence time estimates under the model IIM_3_

Event	Time since occurrence	95% confidence intervals	
		Wald	Profile likelihood
Onset of speciation $\left(t_{0}\right)$	3.624	(3.559, 3.689)	(3.561, 3.691)
Complete isolation $\left(t_{1}\right)$	1.503	(1.419, 1.588)	(1.419, 1.587)

Divergence time estimates for the data of Wang and Hey (2010), given in millions of years ago. Values shown are the converted estimates of $\tau_{0}$ and $\tau_{1}$ (see Figure 1).

**Table 6:** Converted migration rates under the model IIM_3_

Migration parameter	Point estimate	95% confidence intervals	
		Wald	Profile likelihood
Migration rate $\left(q_{1}\right)$	8.8E−09	(1.1E-10, 1.8E−08)	(3.2E−09, 2.4E−08)
Number of migrant sequences $\left(s_{1}\right)$	0.064	(0.001, 0.127)	(0.023, 0.172)

Converted migration rates for the data of Wang and Hey (2010). Values shown refer to forward-in-time parameters: $q_{1}$ is the fraction of subpopulation 1 (*D. simulans*) that migrates to subpopulation 2 (*D. melanogaster*) in each generation, during the period of gene flow; $s_{1}$ is the number of sequences migrating from subpopulation 1 to subpopulation 2 in each generation, during the period of gene flow.

If *g* and $\hat{\mu }$ are treated as constants, then each of the estimators just given can be expressed as a constant times a product—or a ratio—of the estimators of nonconverted parameters. For example, we have that

$${\hat{q}}_{1}=\hat{\mu }\times \frac{{\hat{M}}_{2}\hat{b}}{\hat{\theta }}=\text{constant}\times \frac{{\hat{M}}_{2}\hat{b}}{\hat{\theta }},$$

and

$${\hat{N}}_{a}=\frac{{\hat{\theta }}_{a}}{4\hat{\mu }}=\text{constant}\times {\hat{\theta }}_{a}.$$

Suppose the IIM_3_ model is reparameterized in terms of

$$\varphi ={\left(\theta_{a}\;\theta \;\theta_{b}\;\theta_{c_{1}}\;\theta_{c_{2}}\;T_{1}\;T_{1}+V\;M_{2}b/\theta \right)}^{T},$$

and $\hat{\varphi }$ denotes the maximum-likelihood estimator of φ. Then the estimator ${\hat{\varphi }}_{c}$ of the vector of converted parameters

$$\varphi_{c}={\left(N_{a}\;N\;N_{b}\;N_{c_{1}}\;N_{c_{2}}\;t_{1}\;t_{0}\;q_{1}\right)}^{T},$$

can be written as ${\hat{\varphi }}_{c}=W\hat{\varphi },$ where W is a diagonal matrix. The random vector $\hat{\varphi }$ is a maximum-likelihood estimator (of a reparameterized model). Hence, for a large enough sample size, its distribution is approximately multivariate Gaussian, with some covariance matrix ∑, and the distribution of ${\hat{\varphi }}_{c}$ is approximately multivariate Gaussian with covariance matrix $W\sum W^{T}.$ To calculate the Wald confidence intervals of Table 4, Table 5, and Table 6, we used the inverse of the observed Fisher information as an estimate of ∑. An estimate of $W\sum W^{T}$ followed trivially.

Profile likelihood confidence intervals were also computed for the parameterization $\varphi ={\left(\theta_{a}\;,\ldots ,\;M_{2}b/\theta \right)}^{T}.$ Then, if $\hat{u}$ $\left(\text{or }\hat{l}\right)$ is the vector of estimated upper (or lower) bounds for the parameters in φ, $W\hat{u}$ $\left(\text{or }W\hat{l}\right)$ will be the vector of estimated upper (or lower) bounds for the converted parameters. This follows from the likelihood-ratio invariance—see, for example, Pawitan (2001, pp. 47–48). Confidence intervals for the converted migration parameter $s_{1}$ (rather than $q_{1}$ in the procedure above) were obtained analogously, using a slightly different reparameterization of the IIM_3_ model.

## Discussion

### Notes on our method and results

We have described a fast method to fit the IIM model to large data sets of pairwise differences at a large number of independent loci. This method relies essentially on the eigen-decomposition of the generator matrix of the process during the migration stage of the model: for each set of parameter values, the computation of the likelihood involves this decomposition. Nevertheless, the whole process of estimation takes no more than a couple of minutes for a data set of tens of thousands of loci such as that of Wang and Hey (2010), and it does not require high-performance computing resources. The implementation of the simpler IIM model of Wilkinson-Herbots (2012), with R code provided in Wilkinson-Herbots (2015), is even faster than the more general method presented here, since it makes use of a fully analytical expression for the likelihood (avoiding the need for eigen-decomposition of the generator matrix); but it relies on two assumptions which we have dropped here, and which are typically unrealistic for real species: the symmetry of migration rates and the equality of subpopulation sizes during the gene flow period.

Due to the number of parameters, it is not feasible to assess the performance of our method systematically over every region of the parameter space. However, our experience with simulated data sets suggests that there are two cases in which the variances of some estimators become inflated, in particular the variances of the estimators associated with the gene flow period $({\hat{M}}_{1},$ ${\hat{M}}_{2},$ $\hat{\theta },$ ${\hat{\theta }}_{b},$ and $\hat{V}).$ One of such cases arises whenever *V* is very small or $T_{1}$ is very large, making it very unlikely that the genealogy of a pair of sequences under the IIM model is affected by events that occurred during the gene flow period. The second case arises when the values of the scaled migration rates are greater than one, so that the two subpopulations during the period of gene flow resemble a single panmictic population. In either of these cases, the very process of model fitting can become unstable, that is, the algorithm of maximization of the likelihood may have difficulty converging.

Problems can also arise if the number of loci is insufficient. The simulation study in the *Simulated data* section suggests that convergence to sensible parameter estimates is still possible for a sample size of 8000 loci. However, when we fitted the full IIM model to a simulated sample of 4000 loci (results not shown), outliers started to emerge. It should also be noted that for sample sizes of just a few thousand loci, the distribution of migration rate estimates is still far from Gaussian (Figure 6). In such cases, computation of confidence intervals should be based on bootstrap methods or on the likelihood (profile likelihood confidence intervals) rather than on the Hessian (Wald confidence intervals). How many loci are needed to obtain good estimates and confidence intervals will also depend on the region of the parameter space concerned.

It is not the goal of this article to draw conclusions regarding the evolutionary history of *Drosophila* species. We used the data of Wang and Hey (2010) with the sole objective of demonstrating that our method can be applied efficiently and accurately to real data. In Table 7, we list both our estimates and those of Wang and Hey (2010) for a six-parameter isolation-with-migration model (the IM_1_ model—see Figure 7). The same table contains the estimates for our best-fitting IIM model. Our parameter estimates for the IM model agree well with those of Wang and Hey (2010). The reason that they do not match exactly lies in the fact that we have omitted the “screening procedure” described in Wang and Hey (2010) and have therefore not excluded some of the most divergent sequences in the data set. It should also be borne in mind that our model of mutation is the infinite-sites model, whereas Wang and Hey (2010) have worked with the Jukes–Cantor model. Furthermore, our choice of sequence pairs was somewhat different: Wang and Hey (2010) randomly selected a pair of sequences at each locus, whereas we followed the procedure described in the *Maximum-likelihood estimation* section.

**Table 7:** Comparison of converted estimates obtained with IM and IIM models

	$IM_{\text{wh}}$	IM_1_	IIM_3_
Time since onset of speciation	3.040	3.240	3.624
Time since isolation	—	—	1.503
Size of ancestral population	3.060	4.310	3.549
Current size of *D. simulans* population	5.990	6.120	7.182
Current size of *D. melanogaster* population	2.440	2.700	2.871
Size of *D. simulans* population during IIM gene flow period	—	—	3.640
Size of *D. melanogaster* population during IIM gene flow period	—	—	2.092
Migration rate (*D. simulans →* *D. melanogaster*)	0.013	0.012	0.064
Migration rate (*D. melanogaster→* *D. simulans*)	0.000	0.000	—

Times are given in millions of years; population sizes are given in millions of individuals; the migration rates stated represent the number of sequences that migrate per generation, forward in time. The model $IM_{\text{wh}}$ is the IM model fitted by Wang and Hey (2010).

There are some otable differences between the estimates for both IM models and those for the IIM model: under the IIM model, the process of speciation is estimated to have started earlier (3.6 MYA instead of 3.0 or 3.2 MYA), to have reached complete isolation before the present time (1.5 MYA), and to have a higher rate of gene flow (0.064 sequences per generation instead of 0.013 or 0.012 sequences) during a shorter period of time (2.1 MY of gene flow instead of 3.0 or 3.2 MY). As might be expected, the estimates of each descendant population size (*D. simulans* and *D. melanogaster*) in the IM models lie in between the estimates of the corresponding current population size and its size during the gene flow period in the IIM model.

The method we used assumes that relative mutation rates are known (see *The likelihood of a multilocus data set*). In reality, we must deal with estimates of these rates, and this introduces additional uncertainty which is not reflected in the standard errors and confidence intervals obtained. In principle, this uncertainty can be reduced by increasing the number of ingroup and outgroup sequences used to compute the average number of pairwise differences at each locus in Equation 14. Ideally, estimates of the relative mutation rates should be based on outgroup species only (Wang and Hey 2010) to avoid any dependence between the estimates of relative mutation rates and the observations on ingroup pairwise differences, but this was not possible here since the Wang and Hey (2010) data included exactly one outgroup sequence for each locus.

### Violation of assumptions

Some assumptions of the IIM model in this article, such as the infinite-sites assumption and the assumption of free recombination between loci and no recombination within loci, may not be sensible for some real data sets. The appropriateness of other assumptions, for example those regarding the constant size of populations or the constant rate of gene flow, will depend on the actual evolutionary history of the species or populations involved. While a systematic, in-depth robustness analysis of our method (similar to, for example, the robustness studies by Becquet and Przeworski 2009 and Strasburg and Rieseberg 2010 for commonly used IM methods) is beyond the scope of this article, we will in this section informally examine the impact of possible violations of some of the main assumptions made.

#### Misspecification of the demographic model:

**Figure 9:**
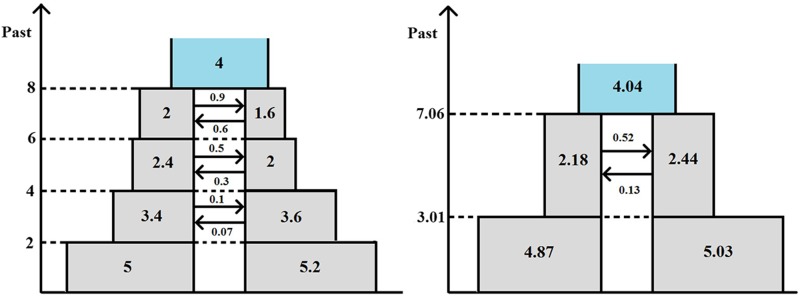
Violation of demographic assumptions. Left-hand-side diagram: true model. Right-hand-side diagram: best-fitting IIM model. Divergence times are measured by twice the expected number of mutations per sequence, population sizes are represented by scaled mutation rates, and rates of gene flow by scaled migration rates.

To explore the potential effect of misspecification of the demographic model on inference accuracy, we first simulated 20 data sets of 40,000 loci each from a somewhat more complex evolutionary scenario, depicted in the left-hand side diagram of Figure 9, where subpopulation sizes gradually increase and gene flow gradually declines. The precise parameter values assumed for the true model were chosen arbitrarily and are shown in the left-hand side diagram; in accordance with the reparameterization used in *Simulated data*, divergence times are measured on a mutational scale by twice the expected number of mutations per sequence (as an average over all loci), population sizes are represented by scaled mutation rates, and rates of gene flow by scaled migration rates. We then applied our method to fit isolation, IM, and IIM models to each of the simulated data sets and selected the best-fitting model by means of likelihood-ratio tests—for each of the 20 data sets generated this was found to be the full IIM model. The average point estimates obtained for each parameter are shown on the right-hand-side diagram of Figure 9. In each diagram, the widths of the boxes are proportional to the population sizes and the heights are proportional to the durations of the time periods concerned. It is readily seen that the IIM model reflects the dynamics of the true model quite well. Population sizes, migration rates, and splitting times are all estimated at intermediate values.

**Figure 10:**
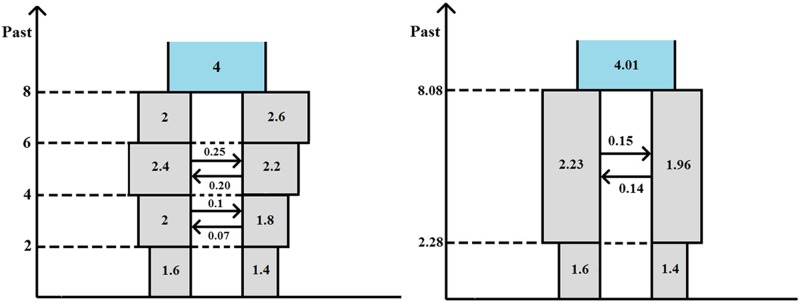
Violation of demographic assumptions. Left-hand-side diagram: true model. Right-hand-side diagram: best-fitting IIM model. Divergence times are measured by twice the expected number of mutations per sequence, population sizes are represented by scaled mutation rates, and rates of gene flow by scaled migration rates.

We also repeated the simulation and estimation procedure for an evolutionary scenario involving a period of secondary gene flow, depicted in the left-hand side diagram of Figure 10. Again, for each of the 20 simulated data sets, the full IIM model provides the best fit among the models considered (isolation, IM, and IIM). Comparing the two diagrams in Figure 10 (where the IIM parameter values in the right-hand-side diagram are again the averages of the estimates obtained for the 20 simulated data sets), we see that the IIM model obtained provides a reasonable approximation to the true model, though of course our method did not detect the initial period of isolation as this feature was not included in the set of models fitted. The estimates of the time since the onset of speciation and the time since complete isolation are, on average, close to the true values in this case. The average estimates of the migration rate and population size parameters are again at intermediate values, compared to the range of true values over time.

#### Intralocus recombination:

In common with other methods mentioned in this article (for example, Wang and Hey 2010; Lohse *et al.* 2011), our method assumes that there is no recombination within loci and free recombination between loci. The first of these two assumptions is the most important one, without which our method would not be valid. Recombination within loci mixes up the genealogies of DNA sequences on which our method relies, making pairs of sequences more equidistant: intralocus recombination does not affect the mean number of segregating sites in a pair of sequences but the *variance* decreases with increasing recombination (Griffiths 1981; Hudson 1983; Schierup and Hein 2000), resulting in data sets which contain more intermediate values and fewer extreme values. This can be expected to lead to overestimation of the current population sizes and underestimation of the ancestral population size, while the effect on estimates of the other parameters is intuitively somewhat less obvious. The impact of intralocus recombination on the variance of the number of pairwise differences, and hence on the accuracy of our method, may be expected to be less severe in cases of recombination rate heterogeneity within loci (see figure 1 in Hudson 1983, for the extreme case of recombination hotspots separating completely linked regions).

A simulation study by Strasburg and Rieseberg (2010) found that even relatively low levels of intralocus recombination can cause substantial bias in estimates of the IM model parameters obtained using the program *IMa* (Hey and Nielsen 2007), with highest posterior density intervals failing to contain the true parameter values far more often than would be expected by chance. In IM simulations allowing a minimal but realistic amount of intralocus recombination, Lohse *et al.* (2016) found that the bias in their parameter estimates was small. Although our method and model are different from those of Hey and Nielsen (2007) and Lohse *et al.* (2016), the effect of recombination on the underlying genealogies remains the same, and therefore similar biases will occur if the assumption of no intralocus recombination is violated.

For the *Drosophila* data considered in this article, Wang and Hey (2010) assessed the impact of potential intralocus recombination on their estimates of the parameters of an IM model by comparison with the estimates obtained from the same sequences but halved in length (*i.e.*, approximately halving the expected number of intralocus recombination events). Their estimates of the ancestral population size and the migration rate from the half-length data were ∼30% larger than those from the full-length data, while the differences for the other parameter estimates were small. In the same spirit, we repeated our previous analysis of the *Drosophila* data but now using the trimmed version of the Wang subset prepared by Lohse *et al.* (2011), in which the average locus length was reduced by approximately a factor of 3; the Hutter subset (∼1% of the total number of loci) was retained in its entirety as we could not afford to further reduce this already very small data set of *D. melanogaster* pairs. Applying the estimation and model selection procedures described in *Drosophila DNA sequence data* to this trimmed version of the data, the likelihood-ratio test of the models IIM_1_ *vs.* IIM_2_ was no longer significant, *i.e.*, there was no longer significant evidence of an increase in population size at time $T_{1},$ and the best-fitting model was a unidirectional version of IIM_1_ (*i.e.*, with $M_{1}=0).$

Table 8 shows the estimates obtained from the trimmed data; the estimates obtained earlier in this article from the full data are also listed again for comparison. In line with our expectations regarding the potential effect of intralocus recombination, it is seen that the full data gave a larger estimate of the current population size of *D. simulans* and a smaller estimate of the ancestral population size; the estimated size of *D. simulans* during the gene flow stage was also smaller than that obtained from the trimmed data. The estimated time since the onset of speciation is nearly identical for the two data sets, but the full data placed the end of gene flow substantially further back into the past (1.5 MYA compared to 0.93 MYA) and estimated a somewhat higher number of migrant sequences per generation (0.064 compared to 0.051) during a shorter period of gene flow (2.12 MY compared to 2.68 MY). This suggests that, in addition to the impact on population size estimates already discussed, intralocus recombination may lead to an overestimate of the time since the end of gene flow in an IIM model and (possibly as a consequence) an overestimate of the migration rate. Nevertheless, for both versions of the *Drosophila* data, the likelihood-ratio tests of nonzero migration rate and nonzero time since the end of gene flow were significant.

**Table 8:** Converted estimates for full sequences and trimmed sequences

	Trimmed		Full
	$\mathrm{II}M_{1}^{*}$	IIM_3_	$\mathrm{II}M_{3}^{*}$
Time since onset of speciation	3.614	3.634	3.624
Time since isolation	0.934	0.997	1.503
Size of ancestral population	4.264	4.237	3.549
Current size of *D. simulans* population	—	6.024	7.182
Current size of *D. melanogaster* population	—	2.984	2.871
Size of *D. simulans* population during gene flow	—	5.956	3.640
Size of *D. melanogaster* population during gene flow	—	1.891	2.092
Size of *D. simulans* population	5.998	—	—
Size of *D. melanogaster* population	2.795	—	—
Migration rate (*D. simulans → D. melanogaster*)	0.051	0.038	0.064
Migration rate (*D. melanogaster →* *D*. *simulans*)	0.000	0.000	0.000

Converted estimates for the data of Wang and Hey (2010). Times are given in millions of years; population sizes are given in millions of individuals; the migration rates stated represent the number of sequences that migrate per generation, forward in time. The best-fitting model for each data set is marked with an *.

The above considerations imply that, when preparing data for use with our method (or any other method relying on the assumption of no intralocus recombination), loci should be chosen carefully to try to keep the amount of intralocus recombination negligible, and some caution may be needed in the interpretation of results. For data sets showing signs of recombination within loci, it may be possible to reduce its effect by trimming or breaking up such loci to form shorter, apparently nonrecombining segments of DNA sequence (Hey and Nielsen 2004; Strasburg and Rieseberg 2010). An extension of our method to account for recombination within loci would be of interest but is challenging. An extension to a finite-sites model for use with shorter fragments of DNA sequence would also be of interest—such an extension is relatively straightforward but is yet to be implemented in our method (but see Wang and Hey 2010 and Andersen *et al.* 2014 for the IM model).

#### Linkage disequilibrium:

If the assumption of free recombination between loci does not hold, then loci are not independent, in which case the likelihood in Equation 13 is in fact a composite marginal likelihood (also called the “independence likelihood” in Chandler and Bate 2007) rather than an ordinary full likelihood (see Varin 2008 for an overview of composite marginal likelihood methods; see also the discussion of Lohse *et al.* 2016). Statistical theory indicates that in that case, the maximum composite likelihood estimator (MCLE) is still consistent (Cox and Reid 2004; Wiuf 2006, with some minor modifications to account for our slightly different assumptions; Varin 2008), provided the relative mutation rates at the different loci are bounded. Thus, if linkage between loci cannot be ignored, the MCLE of the parameters of the IIM model obtained with our method will still be approximately unbiased if the number of loci is sufficiently large, and if all our other assumptions hold (including the assumption of no recombination within loci). However, if linkage between loci is not negligible, then standard errors and confidence intervals computed using the observed Fisher information (as was done in the *Results* section) will underestimate the true uncertainty about the parameter estimates obtained (Baird 2015); instead, standard errors and confidence intervals should be based on an estimate of the Godambe information (Godambe 1960). For a data set made up of a single string of correlated loci, or a small number of such strings, obtaining an accurate estimate of the Godambe information presents some difficulties (see Varin 2008 and Varin *et al.* 2011 for a discussion and some possible strategies). A much simpler situation arises if the data consist of a sufficiently large number of “clusters” of loci, where loci within clusters are correlated but where different clusters can be considered independent. This may be the case, for example, if different clusters of loci are chosen from different chromosomes, or are separated by recombination hotspots or by a large enough distance along the genome. For such data, an empirical estimate of the Godambe information can easily be computed as described in Chandler and Bate (2007) or Varin (2008).

To try to quantify the effect of linkage on the standard errors of the IIM parameter estimates, we conducted the following analysis of a suitable subset of the Wang and Hey (2010) data. We partitioned the 30,247 loci of the Wang subset into blocks of 100 consecutive loci and discarded every other block, so that 151 blocks were retained of 100 loci each. Since the individual loci are ∼500 bp in length and separated by at least 2 kb, this leaves a distance of at least 0.25 Mb between different blocks, and we can reasonably assume that any effect of linkage between blocks of loci this far apart is negligible compared to that within blocks. In the Hutter subset, the distance between consecutive loci is on average ∼50 kb, and we retained these 378 loci to enable estimation of the *D. melanogaster* population size parameters. To examine the effect of linkage, we analyzed this reduced data set in two ways to compare the results: (i) assuming that loci are independent; and (ii) accounting for any linkage between loci within blocks, *i.e.*, accounting for the bulk of the linkage in the data. In case (i), the model selection procedure described in *Model selection* was carried out on the reduced data set. As was the case for the full data, the model IIM_3_ also provided the best fit by far for the reduced data set. The *P*-values computed as part of the model selection procedure were all <10^−42^ and are shown in Table 9. The parameter estimates for the best-fitting model, IIM_3_, are shown in Table 10 and are very close to the estimates obtained from the full Wang and Hey (2010) data (see Table 3). Standard errors of the parameter estimates, based on the Fisher information (computed using the inverted Hessian matrix as described in *Confidence intervals for the selected model*), are also shown in Table 10 for the reduced data set. As expected, these standard errors are larger than those for the full data set by a factor of approximately $\sqrt{2},$ except those of the *D. melanogaster* population size parameters, which are largely unchanged. In case (ii), to account for any linkage within blocks of loci, both the model selection procedure and the computation of standard errors were performed using theoretical results for composite marginal likelihoods. The hypothesis tests in the model selection procedure were carried out using result 3.5 and approximation 3.6 of Jesus and Chandler (2011), by which the null distribution of the composite likelihood-ratio test statistic is approximated by a scaled and shifted $\chi^{2}$ distribution (see also the comments regarding the distribution of the independence likelihood-ratio test statistic in Chandler and Bate (2007), pp.170–171). The *P*-values obtained in this way for the tests in the model selection procedure are shown in Table 9. As expected, these *P*-values are not as small as those obtained when ignoring linkage, and in fact they differ by many orders of magnitude. Nevertheless, these *P*-values are all still smaller than $10^{-20},$ and the model IIM_3_ still gives by far the best fit for the reduced Wang and Hey (2010) data (note however that, to the best of our knowledge, it has not been established in the literature whether the approximate null distribution used for the composite likelihood-ratio test statistic is still conservative in the case of tests involving parameters on the boundary, although this would seem plausible). Standard errors of the parameter estimates of the IIM_3_ model were computed by obtaining an empirical estimate of the inverse of the Godambe information matrix using the method for clustered data described in Chandler and Bate (2007): the covariance matrix of the score vector (the vector of partial derivatives of the log-likelihood) was estimated by

**Table 9:** *P*-values for (composite) likelihood-ratio tests in model selection

**$H_{0}$**	**$H_{1}$**	*P*-values	
		$\chi^{2}$ null distribution*^(i)^*	Robust null distribution*^(ii)^*
ISO	IM_1_	2.60 E−129	1.39 E−110
IM_1_	IIM_1_	8.40 E−57	2.11 E−21
IIM_1_	IIM_2_	1.62 E−43	7.86 E−28

Results for the reduced version of the data of Wang and Hey (2010).

(i) The usual $\chi^{2}$ distribution with the appropriate number of degrees of freedom was used as the null distribution.

(ii) The null distribution used is a scaled and shifted $\chi^{2}$ distribution (Jesus and Chandler 2011, equation 3.6).

**Table 10:** Point estimates and estimated standard errors under the model IIM_3_

Parameter	Estimate	Standard errors	
		Fisher*^(i)^*	Godambe*^(ii)^*
$\theta_{a}$	3.217	0.130	0.146
*θ*	3.259	0.155	0.168
$\theta_{b}$	1.934	0.998	1.251
$\theta_{c_{1}}$	6.833	0.161	0.271
$\theta_{c_{2}}$	2.643	0.174	0.182
$T_{1}$	7.118	0.273	0.435
*V*	9.826	0.228	0.286
$M_{2}$	0.250	0.026	0.035

Results for the reduced version of the data of Wang and Hey (2010). “Fisher” and “Godambe” standard errors are based on the observed Fisher and on the estimated Godambe information matrices, respectively.

$$\hat{V}=\sum_{j}U_{j}U_{j}^{\prime },$$

where the vector $U_{j}$ is the score of the *j*th block of loci, evaluated at the MCLE, and the sum is over all blocks; an estimate of the inverse of the Godambe information matrix (also referred to as the “robust” variance estimator) was then computed as

$${\hat{G}}^{-1}={\hat{H}}^{-1}\hat{V}{\hat{H}}^{-1},$$

where **H** is the Hessian matrix. The resulting standard errors are shown in the right-hand column of Table 10. It is seen that, on average, the standard errors based on the Fisher information account for ∼80% of the uncertainty given by the robust standard errors, though this percentage is different for different parameters. The strongest impact is on the standard error of $\theta_{c_{1}}$ (the “current size” parameter of *D. simulans*), for which the standard error ignoring linkage is only 59% of that which does account for linkage between loci within blocks—one would indeed expect the impact of linkage to be strongest on the standard errors of parameters relating to more recent events, as a shorter time allows less opportunity for recombination between loci (no such effect is seen on the standard error of $\theta_{c_{2}}$ as we continued to treat the Hutter subset as independent loci). To compute standard errors of the parameter estimates obtained from the full Wang and Hey (2010) data, it may be possible to obtain an estimate of the covariance matrix of the score vector, and hence of the Godambe information matrix, by using the method of “window subsampling” (Heagerty and Lele 1998) whereby the data are divided into pseudo-independent subregions, but this would require further investigation. An alternative method to account for linkage disequilibrium is by means of a parametric bootstrap (for example, Lohse *et al.* 2016), but this is computationally intensive and the results will inevitably depend on the recombination rate assumed, and on any other assumptions made such as homogeneity of the recombination rate along the genome.

The robust standard errors in the right-hand column of Table 10 were derived by accounting for linkage while assuming that all our other assumptions hold. If the latter is not the case, then the individual factors in Equation 13 may be misspecified so that their product no longer defines a composite marginal likelihood. Instead, the derivative of its logarithm can be regarded as an “estimating function” and the corresponding statistical theory applied. In that case, our robust calculations of standard errors and *P*-values in (ii) above still apply (Jesus and Chandler 2011, Section 3), so that the results in the right-hand columns of Table 9 and Table 10 are still valid. Thus the differences between the left- and right-hand columns of standard errors and *P*-values in Table 9 and Table 10 should be interpreted as *upper bounds* on the impact of linkage, since these differences may in part be due to other forms of model misspecification, including model misspecification from any of the potential sources discussed above: inaccurate estimates of the relative mutation rates, misspecification of the mutation model, misspecification of the demographic model, and intralocus recombination.

## Supplementary Material

Supplemental material is available online at www.genetics.org/lookup/suppl/doi:10.1534/genetics.116.188060/-/DC1.

## Appendix A: Proof of Equation 8:

This proof has three parts. *Part (i)* proves the result under two assumptions: (a) $Q_{mig}$ has three strictly negative eigenvalues and one zero eigenvalue, all of them real; and (b) $Q_{mig}$ is diagonalizable. *Part (ii)* proves assumption (a). *Part (iii)* proves assumption (b). To simplify the notation, we denote $Q_{mig}$ by Q throughout the proof.

### Part (i)

Consider the continuous-time Markov chain defined by the matrix Q. Let $P_{ij}\left(t\right),$ the (i,j) entry of the matrix P(t), be the probability that the process is in state *j* at time *t* into the past, given that the process starts in state *i*. P(t) can be calculated by solving the following initial value problem:

$$\begin{array}{l} P^{\prime }\left(t\right)=P\left(t\right)Q;\\ P\left(0\right)=I_{4}, \end{array}$$

where $I_{4}$ is the four-by-four identity matrix. Under the assumptions that Q is diagonalizable and that its eigenvalues are real, the solution to this initial value problem is given by:

$$\begin{array}{c} P\left(t\right)=P\left(0\right)e^{Qt}\\ =V^{-1}e^{Bt}V, \end{array}$$

where B denotes the diagonal matrix containing the real eigenvalues $\beta_{j},$ j∈{1,2,3,4}, of Q; and V is the matrix of left eigenvectors of Q. Note that $P_{i4}\left(t\right)$ is the probability that the process has reached coalescence by time *t*, if it started in state *i*. In other words, it is the CDF of $T_{\mathrm{mig}}^{\left(i\right)}:$

$$P_{i4}\left(t\right)=F_{\mathrm{mig}}^{\left(i\right)}\left(t\right)=v_{i.}^{-1}e^{Bt}v_{.4},$$

where $v_{i.}^{-1}$ is the $i^{\text{th}}$ row vector of $V^{-1},$ and $v_{.4}$ the fourth column vector of V. Differentiating, we get the PDF:

$$\begin{array}{c} f_{\mathrm{mig}}^{\left(i\right)}\left(t\right)=v_{i.}^{-1}Be^{Bt}v_{.4}\\ =\sum_{j=1}^{4}V_{ij}^{-1}V_{j4}\beta_{j}e^{\beta_{j}t}. \end{array}$$

If we denote the eigenvalue equal to zero by $\beta_{4},$ and the remaining eigenvalues are strictly negative, this PDF can be written as a linear combination of exponential densities:

$$f_{\mathrm{mig}}^{\left(i\right)}\left(t\right)=-\sum_{j=1}^{3}V_{ij}^{-1}V_{j4}\lambda_{j}e^{-\lambda_{j}t},$$ (A1)

where $\lambda_{j}=\left|\beta_{j}\right|$ for j∈{1,2,3}.

### Part (ii)

As Q is given by Equation 3, its characteristic polynomial, $P_{Q}\left(\beta \right),$ is of the form $\beta \times P_{Q^{\left(r\right)}}\left(\beta \right),$ where $Q^{\left(r\right)}$ is the three-by-three upper-left submatrix of Q, that is:

$$Q^{\left(r\right)}=\begin{array}{c} \left[\begin{array}{ccc} -\left(1+M_{1}\right) & M_{1} & 0\\ M_{2}/2 & -\left(M_{1}+M_{2}\right)/2 & M_{1}/2\\ 0 & M_{2} & -\left(1/b+M_{2}\right) \end{array}\right]. \end{array}$$

Thus the eigenvalues of Q are the solutions to $\beta \times P_{Q^{\left(r\right)}}\left(\beta \right)=0.$ Consequently, one of them is zero $\left(\beta_{4},\text{ say}\right)$ and the remaining three eigenvalues are also eigenvalues of $Q^{\left(r\right)}.$

Now consider the similarity transformation

$$S=DQ^{\left(r\right)}D^{-1}=\left[\begin{array}{ccc} -\left(1+M_{1}\right) & \sqrt{\frac{M_{1}M_{2}}{2}} & 0\\ \sqrt{\frac{M_{1}M_{2}}{2}} & -\left(M_{1}+M_{2}\right)/2 & \sqrt{\frac{M_{1}M_{2}}{2}}\\ 0 & \sqrt{\frac{M_{1}M_{2}}{2}} & -\left(1/b+M_{2}\right) \end{array}\right],$$

$$\text{where }D=\left[\begin{array}{ccc} \sqrt{\frac{M_{2}}{2M_{1}}} & 0 & 0\\ 0 & 1 & 0\\ 0 & 0 & \sqrt{\frac{M_{1}}{2M_{2}}} \end{array}\right].$$

Because S is a symmetric matrix, its eigenvalues are real. Therefore, all the eigenvalues of $Q^{\left(r\right)}$ are real (a similarity transformation does not change the eigenvalues). S is also a negative definite matrix, since its first, second, and third upper-left determinants are respectively negative, positive, and negative. Hence its eigenvalues are all strictly negative, and so are the eigenvalues of $Q^{\left(r\right)}.$ Hence Q has one zero eigenvalue $\left(\beta_{4}\right)$ and three real, strictly negative eigenvalues $\left(\beta_{1},\;\beta_{2}\text{ and }\beta_{3}\right).$

### Part (iii)

Being a symmetric matrix, S has three independent eigenvectors. A similarity transformation preserves the number of independent eigenvectors, so $Q^{\left(r\right)}$ has three independent eigenvectors as well. We denote by $V^{\left(r\right)}$ the matrix whose rows are the left eigenvectors of $Q^{\left(r\right)}.$

By definition, any left eigenvector $v_{j.}$ of Q satisfies the system of equations $x\left(Q-I\beta_{j}\right)=0,$ where $x=\left[x_{1}\;x_{2}\;x_{3}\;x_{4}\right].$. The first three linear equations of this system are identical to $x^{\left(r\right)}\left[Q^{\left(r\right)}-I\beta_{j}\right]=0,$ for j∈{1,2,3} and $x^{\left(r\right)}=\left[x_{1}\;x_{2}\;x_{3}\right],$ which is solved by $x^{\left(r\right)}=v_{j.}^{\left(r\right)}.$ So this implies that, for $\beta_{j}\in \left\{\beta_{1},\beta_{2},\beta_{3}\right\},$ any row vector x in ${\mathbb{R}}^{4}$ that has $v_{j.}^{\left(r\right)}$ as its first three elements will solve the first three equations of the system, whatever the value of $x_{4}.$ If $x_{4}=\left[V_{j1}^{\left(r\right)}+\frac{1}{b}V_{j3}^{\left(r\right)}\right]/\beta_{j},$ that vector will be an eigenvector of Q, because it also solves the fourth equation of the system:

$$\begin{array}{l} \left[\begin{array}{cc} \text{——–}v_{j.}^{\left(r\right)}\text{——–} & \frac{V_{j1}^{\left(r\right)}+\frac{1}{b}V_{j3}^{\left(r\right)}}{\beta_{j}} \end{array}\right]\begin{array}{c} \left[\begin{array}{cccc} -\left(1+M_{1}\right)-\beta_{j} & M_{1} & 0 & 1\\ \frac{M_{2}}{2} & -\frac{\left(M_{1}+M_{2}\right)}{2}-\beta_{j} & \frac{M_{1}}{2} & 0\\ 0 & M_{2} & -\left(\frac{1}{b}+M_{2}\right)-\beta_{j} & \frac{1}{b}\\ 0 & 0 & 0 & -\beta_{j} \end{array}\right] \end{array}\\ =\left[\begin{array}{cccc} 0 & 0 & 0 & 0 \end{array}\right], \end{array}$$

for $\beta_{j}\in \left\{\beta_{1},\beta_{2},\beta_{3}\right\}.$. For the case of $\beta_{j}=\beta_{4}=0,$ a row eigenvector is [0 0 0 1]. Collecting these row eigenvectors in a single matrix, we get V. So,

$$V=\left\{\begin{array}{cccc} \text{——} & v_{1.}^{\left(r\right)} & \text{——} & \frac{\left[V_{11}^{\left(r\right)}+\frac{1}{b}V_{13}^{\left(r\right)}\right]}{\beta_{1}}\\ \text{——} & v_{2.}^{\left(r\right)} & \text{——} & \frac{\left[V_{21}^{\left(r\right)}+\frac{1}{b}V_{23}^{\left(r\right)}\right]}{\beta_{2}}\\ \text{——} & v_{3.}^{\left(r\right)} & \text{——} & \frac{\left[V_{31}^{\left(r\right)}+\frac{1}{b}V_{33}^{\left(r\right)}\right]}{\beta_{3}}\\ 0 & 0 & 0 & 1 \end{array}\right\}.$$

If the matrix V can be shown to be invertible, then Q is diagonalizable. This will be the case if the system xV=0 can only be solved by x=[0 0 0 0].. Now since the three-by-three upper-left submatrix of V, $V^{\left(r\right)},$ is full-ranked, $x_{1}=x_{2}=x_{3}=0$ is a necessary condition for xV=0. But then $x_{4}=0,$ from the last equation of the system. Thus we have shown that Q is diagonalizable. □

### Migration from Subpopulation 1 to Subpopulation 2 Backward in Time (M_1_ > 0, M_2_ = 0)

Using the derivation procedure described in *Distribution of the time until coalescence under unidirectional gene flow*, *and in the absence of gene flow*, we find that:

$$\begin{array}{c} f_{\mathrm{mig}}^{\left(1\right)}\left(t\right)=\left[\frac{b^{2}M_{1}^{2}}{\left(bM_{1}-2\right)\left(b-1+bM_{1}\right)}\right]\frac{1}{b}e^{-\frac{1}{b}t}+\left[\frac{4M_{1}}{\left(2-bM_{1}\right)\left(2+M_{1}\right)}\right]\frac{M_{1}}{2}e^{-\frac{M_{1}}{2}t}\\ +\left[\frac{1}{\left(1+M_{1}\right)}+\frac{M_{1}^{2}}{\left(2+M_{1}\right)\left(b-1+bM_{1}\right)\left(1+M_{1}\right)}\right]\left(1+M_{1}\right)e^{-\left(1+M_{1}\right)t}\\ f_{\mathrm{mig}}^{\left(2\right)}\left(t\right)=\frac{1}{b}e^{-\left(1/b\right)t}\\ f_{\mathrm{mig}}^{\left(3\right)}\left(t\right)=\left(\frac{bM_{1}}{bM_{1}-2}\right)\frac{1}{b}e^{-\frac{1}{b}t}+\left(\frac{2}{2-bM_{1}}\right)\frac{M_{1}}{2}e^{-\frac{M_{1}}{2}t}. \end{array}$$

As a result, the PDF of the coalescence time of a pair of sequences under the IIM model, $f_{T}^{\left(i\right)}\left(t\right),$ is again given by Equations 9 and 10, now with

$$\lambda =\left[\begin{array}{ccc} \frac{1}{b} & \frac{M_{1}}{2} & 1+M_{1} \end{array}\right]$$

and

$$A=\left[\begin{array}{ccc} \frac{b^{2}M_{1}^{2}}{\left(bM_{1}-2\right)\left(b-1+bM_{1}\right)} & \frac{4M_{1}}{\left(2-bM_{1}\right)\left(2+M_{1}\right)} & \frac{1}{1+M_{1}}+\frac{M_{1}^{2}}{\left(2+M_{1}\right)\left(b-1+bM_{1}\right)\left(1+M_{1}\right)}\\ 1 & 0 & 0\\ \frac{bM_{1}}{bM_{1}-2} & \frac{2}{2-bM_{1}} & 0 \end{array}\right].$$

### Distribution of the Time Until Coalescence Under an IIM Model with M_1_ = M_2_ = 0

In this case, the IIM model reduces to a complete isolation model where both descendant populations may change size at time $\tau_{1}$ into the past. The distribution of the absorption time $T_{\mathrm{mig}}^{\left(i\right)}$ corresponding to $Q_{mig}$ will now be either exponential, if both sampled sequences are from the same subpopulation (*i.e.*, for i∈{1,2}); or coalescence will not be possible at all until the ancestral population is reached, if we take a sequence from each subpopulation (*i.e.*, if *i* = 3). It follows that the PDF of the coalescence time of a pair of sequences in the IIM model is given by Equations 9 and 10 with

$$\lambda =\left[\begin{array}{ccc} 1 & \left(1/\text{b}\right) & 0 \end{array}\right]$$

and

$$A=\left[\begin{array}{ccc} 1 & 0 & 0\\ 0 & 1 & 0\\ 0 & 0 & 1 \end{array}\right].$$

## References

[bib1] Andersen L. Mailund T. Hobolth A., 2014 Efficient computation in the IM model. J. Math. Biol. 68: 1423–1451. 23588350 10.1007/s00285-013-0671-9

[bib2] Baird S. J., 2015 Exploring linkage disequilibrium. Mol. Ecol. Resour. 15: 1017–1019. 26261040 10.1111/1755-0998.12424

[bib3] Becquet C. Przeworski M., 2007 A new approach to estimate parameters of speciation models with application to apes. Genome Res. 17: 1505–1519. 17712021 10.1101/gr.6409707 PMC1987350

[bib4] Becquet C. Przeworski M., 2009 Learning about modes of speciation by computational approaches. Evolution 63: 2547–2562. 19228187 10.1111/j.1558-5646.2009.00662.x

[bib5] Casella G. Berger R., 2001 Statistical Inference, Ed. 2 Duxbury, Belmont, CA.

[bib6] Chandler R. E. Bate S., 2007 Inference for clustered data using the independence loglikelihood. Biometrika 94: 167–183.

[bib7] Cox D. R. Reid N., 2004 A note on pseudolikelihood constructed from marginal densities. Biometrika 91: 729–737.

[bib8] Fisher R. A., 1930 The Genetical Theory of Natural Selection, Ed. 1 Clarendon Press, Oxford.

[bib9] Godambe V. P., 1960 An optimum property of regular maximum likelihood estimation. Ann. Math. Stat. 31: 1208–1211.

[bib10] Griffiths R. C., 1981 The number of heterozygous loci between two randomly chosen completely linked sequences of loci in two subdivided population models. J. Math. Biol. 12: 251–261.

[bib11] Gutenkunst R. N. Hernandez R. D. Williamson S. H. Bustamante C. D., 2009 Inferring the joint demographic history of multiple populations from multidimensional SNP frequency data. PLoS Genet. 5: e1000695. 19851460 10.1371/journal.pgen.1000695 PMC2760211

[bib12] Heagerty P. J. Lele S. R., 1998 A composite likelihood approach to binary spatial data. J. Am. Stat. Assoc. 93: 1099–1111.

[bib13] Hein J. Schierup M. H. Wiuf C., 2005 *Gene Genealogies*, *Variation and Evolution*. Oxford University Press, Oxford.

[bib14] Hey J., 2005 On the number of New World founders: a population genetic portrait of the peopling of the Americas. PLoS Biol. 3: e193. 15898833 10.1371/journal.pbio.0030193 PMC1131883

[bib15] Hey J., 2010 Isolation with migration models for more than two populations. Mol. Biol. Evol. 27: 905–920. 19955477 10.1093/molbev/msp296 PMC2877539

[bib16] Hey J. Nielsen R., 2004 Multilocus methods for estimating population sizes, migration rates and divergence time, with applications to the divergence of *Drosophila pseudoobscura* and *D. persimilis*. Genetics 167: 747–760. 15238526 10.1534/genetics.103.024182 PMC1470901

[bib17] Hey J. Nielsen R., 2007 Integration within the Felsenstein equation for improved Markov chain Monte Carlo methods in population genetics. Proc. Natl. Acad. Sci. USA 104: 2785–2790. 17301231 10.1073/pnas.0611164104 PMC1815259

[bib18] Hudson R. R., 1983 Properties of a neutral allele model with intragenic recombination. Theor. Popul. Biol. 23: 183–201. 6612631 10.1016/0040-5809(83)90013-8

[bib19] Hutter S. Li H. Beisswanger S. De Lorenzo D. Stephan W., 2007 Distinctly different sex ratios in African and European populations of *Drosophila melanogaster* inferred from chromosomewide single nucleotide polymorphism data. Genetics 177: 469–480. 17660560 10.1534/genetics.107.074922 PMC2013676

[bib20] Innan H. Watanabe H., 2006 The effect of gene flow on the coalescent time in the human-chimpanzee ancestral population. Mol. Biol. Evol. 23: 1040–1047. 16495349 10.1093/molbev/msj109

[bib21] Janko, K., J. Pačes, H. Wilkinson-Herbots, R. J. Costa, J. Röslein *et al.*, 2016 Hybrid asexuality as a primary reproductive barrier: on the interconnection between asexuality and speciation. bioRxiv Available at: https://doi.org/10.1101/038299. 10.1111/mec.14377 PMC6849617 28987005

[bib22] Jesus J. Chandler R. E., 2011 Estimating functions and the generalized method of moments. Interface focus 1: 871–885. 23226587 10.1098/rsfs.2011.0057 PMC3262292

[bib23] Kamm J. A. Terhorst J. Song Y. S., 2016 Efficient computation of the joint sample frequency spectra for multiple populations. J. Comput. Graph. Stat. 26: 182–194. 10.1080/10618600.2016.1159212 PMC5319604 28239248

[bib24] Kingman J. F. C., 1982 a The coalescent. Stochastic Process. Appl. 13: 235–248.

[bib25] Kingman J. F. C., 1982 b On the genealogy of large populations. J. Appl. Probab. 19: 27–43.

[bib26] Kopylev L. Sinha B., 2011 On the asymptotic distribution of likelihood ratio test when parameters lie on the boundary. Sankhya B 73: 20–41.

[bib27] Lohse K. Harrison R. J. Barton N. H., 2011 A general method for calculating likelihoods under the coalescent process. Genetics 189: 977–987. 21900266 10.1534/genetics.111.129569 PMC3213358

[bib28] Lohse K. Clarke M. Ritchie M. G. Etges W. J., 2015 Genome-wide tests for introgression between cactophilic *Drosophila* implicate a role of inversions during speciation. Evolution 69: 1178–1190. 25824653 10.1111/evo.12650 PMC5029762

[bib29] Lohse K. Chmelik M. Martin S. H. Barton N. H., 2016 Efficient strategies for calculating blockwise likelihoods under the coalescent. Genetics 202: 775–786. 26715666 10.1534/genetics.115.183814 PMC4788249

[bib30] Mailund T. Halager A. E. Westergaard M. Dutheil J. Y. Munch K., 2012 A new isolation with migration model along complete genomes infers very different divergence processes among closely related great ape species. PLoS Genet. 8: e1003125. 23284294 10.1371/journal.pgen.1003125 PMC3527290

[bib31] Nath H. Griffiths R., 1993 The coalescent in two colonies with symmetric migration. J. Math. Biol. 31: 841–851. 8263428 10.1007/BF00168049

[bib32] Nielsen R. Wakeley J., 2001 Distinguishing migration from isolation: a Markov chain Monte Carlo approach. Genetics 158: 885–896. 11404349 10.1093/genetics/158.2.885 PMC1461674

[bib33] Notohara M., 1990 The coalescent and the genealogical process in geographically structured population. J. Math. Biol. 29: 59–75. 2277236 10.1007/BF00173909

[bib34] Pawitan Y., 2001 In All Likelihood: Statistical Modelling and Inference Using Likelihood. Oxford University Press, Oxford.

[bib35] Pinho C. Hey J., 2010 Divergence with gene flow: models and data. Annu. Rev. Ecol. Evol. Syst. 41: 215–230.

[bib36] Powell J. R., 1997 Progress and Prospects in Evolutionary Biology: The Drosophila Model. Oxford University Press, Oxford.

[bib37] Schierup M. H. Hein J., 2000 Consequences of recombination on traditional phylogenetic analysis. Genetics 156: 879–891. 11014833 10.1093/genetics/156.2.879 PMC1461297

[bib38] Schiffels S. Durbin R., 2014 Inferring human population size and separation history from multiple genome sequences. Nat. Genet. 46: 919–925. 24952747 10.1038/ng.3015 PMC4116295

[bib39] Self S. G. Liang K.-Y., 1987 Asymptotic properties of maximum likelihood estimators and likelihood ratio tests under nonstandard conditions. J. Am. Stat. Assoc. 82: 605–610.

[bib40] Steinrücken M. Kamm J. A. Song Y. S., 2015 Inference of complex population histories using whole-genome sequences from multiple populations. bioRxiv Available at: https://doi.org/10.1101/026591. 10.1073/pnas.1905060116 PMC6708337 31387977

[bib41] Strasburg J. L. Rieseberg L. H., 2010 How robust are “isolation with migration” analyses to violations of the IM model? A simulation study. Mol. Biol. Evol. 27: 297–310. 19793831 10.1093/molbev/msp233 PMC2877552

[bib42] Teshima K. M. Tajima F., 2002 The effect of migration during the divergence. Theor. Popul. Biol. 62: 81–95. 12056866 10.1006/tpbi.2002.1580

[bib43] Varin C., 2008 On composite marginal likelihoods. AStA Adv. Stat. Anal. 92: 1–28.

[bib44] Varin C. Reid N. Firth D., 2011 An overview of composite likelihood methods. Stat. Sin. 21: 5–42.

[bib45] Wakeley J. Hey J., 1997 Estimating ancestral population parameters. Genetics 145: 847–855. 9055093 10.1093/genetics/145.3.847 PMC1207868

[bib46] Wang Y. Hey J., 2010 Estimating divergence parameters with small samples from a large number of loci. Genetics 184: 363–379. 19917765 10.1534/genetics.109.110528 PMC2828718

[bib47] Watterson G., 1975 On the number of segregating sites in genetical models without recombination. Theor. Popul. Biol. 7: 256–276. 1145509 10.1016/0040-5809(75)90020-9

[bib48] Wilkinson-Herbots H. M., 1998 Genealogy and subpopulation differentiation under various models of population structure. J. Math. Biol. 37: 535–585.

[bib49] Wilkinson-Herbots H. M., 2008 The distribution of the coalescence time and the number of pairwise nucleotide differences in the isolation with migration model. Theor. Popul. Biol. 73: 277–288. 18215405 10.1016/j.tpb.2007.11.001

[bib50] Wilkinson-Herbots H. M., 2012 The distribution of the coalescence time and the number of pairwise nucleotide differences in a model of population divergence or speciation with an initial period of gene flow. Theor. Popul. Biol. 82: 92–108. 22687581 10.1016/j.tpb.2012.05.003

[bib51] Wilkinson-Herbots, H. M., 2015 A fast method to estimate speciation parameters in a model of isolation with an initial period of gene flow and to test alternative evolutionary scenarios. arXivAvailable at: https://arxiv.org/abs/1511.05478.

[bib52] Wiuf C., 2006 Consistency of estimators of population scaled parameters using composite likelihood. J. Math. Biol. 53: 821–841. 16960689 10.1007/s00285-006-0031-0

[bib53] Wright S., 1931 Evolution in Mendelian populations. Genetics 16: 97–159. 17246615 10.1093/genetics/16.2.97 PMC1201091

[bib54] Yang Z., 2002 Likelihood and Bayes estimation of ancestral population sizes in hominoids using data from multiple loci. Genetics 162: 1811–1823. 12524351 10.1093/genetics/162.4.1811 PMC1462394

[bib55] Zhu T. Yang Z., 2012 Maximum likelihood implementation of an isolation-with-migration model with three species for testing speciation with gene flow. Mol. Biol. Evol. 29: 3131–3142. 22504520 10.1093/molbev/mss118

